# A Quantitative Proteomic Analysis Uncovers the Relevance of CUL3 in Bladder Cancer Aggressiveness

**DOI:** 10.1371/journal.pone.0053328

**Published:** 2013-01-08

**Authors:** Laura Grau, Jose L. Luque-Garcia, Pilar González-Peramato, Dan Theodorescu, Joan Palou, Jesus M. Fernandez-Gomez, Marta Sánchez-Carbayo

**Affiliations:** 1 Tumor Markers Group, Spanish National Cancer Research Center, Madrid, Spain; 2 Department of Analytical Chemistry, Complutense University of Madrid, Madrid, Spain; 3 Pathology Department, Hospital Universitario La Paz, Madrid, Spain; 4 Mellon Urologic Cancer Institute, University of Virginia, Charlottesville, Virginia, United States of America; 5 Urology Department, Fundacio Puigvert, Barcelona, Spain; 6 Urology Department, Hospital Central de Asturias, Oviedo, Spain; Moffitt Cancer Center, United States of America

## Abstract

To identify aggressiveness-associated molecular mechanisms and biomarker candidates in bladder cancer, we performed a SILAC (Stable Isotope Labelling by Amino acids in Cell culture) proteomic analysis comparing an invasive T24 and an aggressive metastatic derived T24T bladder cancer cell line. A total of 289 proteins were identified differentially expressed between these cells with high confidence. Complementary and validation analyses included comparison of protein SILAC data with mRNA expression ratios obtained from oligonucleotide microarrays, and immunoblotting. Cul3, an overexpressed protein in T24T, involved in the ubiquitination and subsequent proteasomal degradation of target proteins, was selected for further investigation. Functional analyses revealed that Cul3 silencing diminished proliferative, migration and invasive rates of T24T cells, and restored the expression of cytoskeleton proteins identified to be underexpressed in T24T cells by SILAC, such as ezrin, moesin, filamin or caveolin. Cul3 immunohistochemical protein patterns performed on bladder tumours spotted onto tissue microarrays (n = 284), were associated with tumor staging, lymph node metastasis and disease-specific survival. Thus, the SILAC approach identified that Cul3 modulated the aggressive phenotype of T24T cells by modifying the expression of cytoskeleton proteins involved in bladder cancer aggressiveness; and played a biomarker role for bladder cancer progression, nodal metastasis and clinical outcome assessment.

## Introduction

Bladder cancer represents the 4th most common malignancy among men and the 8th most frequent cause of male cancer deaths [1]. Clinically, approximately 75% of transitional cell carcinomas (TCC) are non-muscle invasive (TIS, Ta, and T1), 20% muscle infiltrating (T2–T4), and 5% metastatic at the time of diagnosis [1]. Low-grade tumors are papillary and usually non-invasive, while high-grade tumors can be either papillary or non-papillary, and often invasive. Patients diagnosed with localized TCC have a 5-year survival rate above 90%. However, patients with regional and distant metastatic disease have a 5-year survival rate below 50% and 10%, respectively [1]. Bladder cancer progression follows complex sequential steps, not completely understood [2]–[4]. Differences in aggressiveness behaviour have been described between the invasive T24 bladder cancer cell line and the more aggressive T24T variant that develops metastases after tail vein injection [5]–[9]. Identification of differentially expressed proteins between these cells might uncover molecular mechanisms associated with tumor aggressiveness in vitro potentially leading to metastasis. Proteins participating in such pathways could serve as biomarkers for either early identification of aggressive outcome and/or potentially be therapeutically targetable.

**Figure 1:**
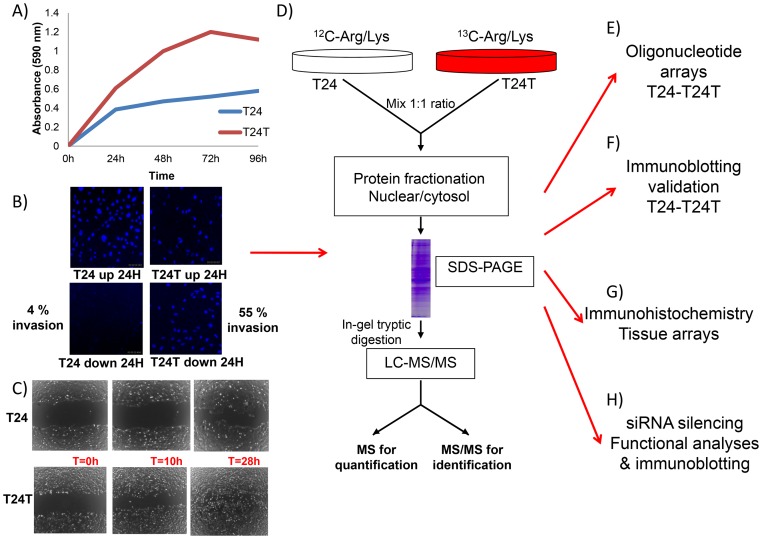
Cell line phenotypes and experimental design. Functional analyses were performed to assess the differential aggressive phenotype of T24 and T24T bladder cancer cells on: **A**) proliferation, **B**) invasion, and **C**) migration. The average of duplicate experiments for each functional assay of these cells at several timepoints is represented in each panel. **D**) Schematic diagram showing the workflow used for the multiplexed SILAC-based experiments. Internal labelling was performed *in vitro*, the protein extracts were fractionated via SDS-PAGE, digested with trypsin in gel, and tryptic digests were analyzed by LC-MS/MS to both identify and quantify the proteins present. **E)** Comparison of the protein changes identified by SILAC was performed with those observed by oligonucleotide arrays. **F**) Validation of the protein changes identified by SILAC in Western blots of protein extracts obtained from T24-T24T cells. **G**) Immunohistochemistry on tissue arrays containing bladder tumors served to validate associations of identified proteins with clinicopathological variables in bladder cancer. **H**) siRNA silencing of identified proteins and subsequent functional analyses and immunoblotting validation served to evaluate the impact of identified candidates on the aggressive phenotype of T24T and the regulation of other differentially expressed proteins identified by SILAC.

Quantitative proteomics contributes to the discovery of candidate disease-specific target and biomarkers. While protein and antibody arrays permit differential quantification of known proteins [10], [11], mass spectrometry techniques lead for protein identification [12]. Stable isotope labelling by amino acids in cell culture (SILAC) involves the addition of (12)C- and (13)C-labeled amino acids to growth media of separately cultured cells, giving rise to cells containing "light" or "heavy" proteins, respectively [12]–[31]. To our knowledge, SILAC has not been reported in bladder cancer. Here, a quantitative proteomic analysis was applied to T24 and T24T cells to identify proteins and pathways related to their differential aggressiveness following our experimental design (Figure 1).

## Materials and Methods

### 1. Functional analysis of T24 and T24T bladder cancer cells

#### Cell culture

T24 was obtained from the American Type Culture Collection and cultured as previously described [32], [33]. T24T was derived from T24 at Dr Theodorescu’s laboratory [5]–[9]. Cells were grown for 4–6 passages and harvested at 75%-90% confluency. Cell pellets were washed three times in cold PBS, and frozen at -20°C before RNA and protein extraction.

### Proliferation assay

1.2x10^4^ cells per well were seeded in 96-well plates in triplicate in DMEM containing 10% FBS. After culturing for 24, 48, 72 and 96 hours, proliferation was measured with the MTT assay (Roche, Mannheim, Germany).

#### Wound healing assay

3.5x10^5^ cells were seeded in 6-well plates, and a wound was made in the monolayer using a sterile pipette tip once the cells reached confluency. Photographs of cells invading the wound were taken at the indicated times.

#### Invasion assay

Cell culture 24-well plates inserts (pore size 8 µm, BD Biosciences, San José, CA) were seeded with 2.5x10^4^ T24 and T24T cells, and also with T24T cells after 24 and 48 hours post-transfection with Cul3 siRNA (50 nM) in 500 µL of DMEM medium with 0.1% FBS in the upper chamber. Medium with 10% FBS (500 µL) was added to the lower chamber as a chemotactic agent. Matrigel invasion chambers (BD) were maintained for 24 hours in a humidified incubator at 37°C, 5% CO_2_ atmosphere. Cells on both sides of the matrigel chamber were fixed with 4% paraformaldehyde for 10 minutes, washed with PBS, stained with 1µg/mL 4'-6-Diamidino-2-phenylindole (DAPI: Sigma, St Louis, MO) for 10 minutes, and analysed by confocal microscopy (Leica TCS-SP5, Wetzlar, Germany). The number of invading cells was assessed with the Imaris software (Bit Plane, Zurich, Switzerland), estimating the percentage of invasion as: number of invading cells/number of total cells×100.

#### Cul3 silencing

Cul3 knocked-down was performed in T24T by transient transfection with Lipofectamine (Invitrogen, Carlsbad, CA) using control (not-targeting) small interfering double-stranded RNA (siRNA) and the smart pool siRNA targeted against Cul3 (both from Dharmacon, Waltham, MA). Cul3 silencing transfectants exposed to 50nM and 100Nm of targeted siRNA were collected at 24h and 48h for proliferation, migration or invasion assays, as described above. Cul3 silencing was confirmed by immunoblotting.

### 2. SILAC protein profiling

#### Cell Culture and Metabolic Labeling

T24 and T24T cells were maintained in lysine and arginine-depleted DMEM (Millipore, Billerica, MA) supplemented with 10% dialyzed FBS (Invitrogen, Carlsbad, CA), 100 units/mL of penicillin/streptomycin (Invitrogen) and either naturally-occurring isotope abundances (“light”) (T24) or stable isotope-labelled (“heavy”) ^13^C_6_ lysine and ^13^C_6_ arginine amino acids (Cambridge Isotope Labs, Andover, MA) (T24T). Culture media were refreshed every 2 days by removing half of the volume present on each plate and replacing it with fresh medium. Cells were grown for at least 6 doublings to allow full incorporation of labelled amino acids. Two large-scale SILAC replicates (2×10^7^ cells per condition) were performed. Complete incorporation of ^13^C-Arg and ^13^C-Lys into T24 and T24T cells after six cell divisions in isotopically heavy medium (direct and reverse labeling) was verified by MS of a protein digest.

#### Protein Fractionation

To reduce the complexity of the sample, a nuclear/cytosol fractionation was performed. Cells were lysed in a lysis buffer (20 mM HEPES, pH 7.0, 10 mM KCl, 2 mM MgCl, 0.5% Nonidet P40, 1 mM Na_3_VO_4_, 1 mM PMSF, 0.15 U mL^-1^ aprotinin) and homogenized by 30 strokes in a Dounce homogenizer. The homogenate was centrifuged at 1,500 g for 5 min to sediment the nuclei. The supernatant was then resedimented at 15,000 g for 5 min, and the resulting supernatant formed the non-nuclear or cytosol fraction. The nuclear pellet was washed three times and resuspended in the same buffer containing 0.5 M NaCl. The extracted material was sedimented at 15,000 g for 10 min and the resulting supernatant was termed the nuclear fraction.

#### SDS-PAGE and in-gel digestion

Proteins in cytosolic and nuclear fractions were separated by SDS-PAGE on 10% SDS-polyacrylamide gels. A total of 80 µg of protein was loaded per lane. After electrophoresis, proteins were visualized by Coomassie blue staining and the gel lane was cut horizontally into 20 sections. Excised gel bands were cut into small pieces and destained in 50∶50 25 mM ammonium bicarbonate/acetonitrile, dehydrated with acetonitrile and dried. Gel pieces were rehydrated with 30 µL of 12.5 ng/mL trypsin solution in 25 mM ammonium bicarbonate and incubated overnight at 37°C. Peptides were extracted using acetonitrile and 5% formic acid, dried by vacuum centrifugation and resuspended in 15 µL of 2% acetonitrile in 0.1% formic acid. All samples were sonicated for 10 min before MS analysis.

#### Nanoflow LC-MS/MS

The peptide mixture from in-gel tryptic digestions (using 30 µL of trypsin at 12.5 ng/mL) was analyzed using nanoflow LC-MS/MS. Peptides were loaded onto a trap column (Reprosil C_18_, 3 µm particle size, 0,3×10 mm, 120 Å pore size, SGE) and then eluted to the analytical column (Acclaim PepMap 100, C_18_, 3 µm particle size, 75 µm×15 cm, 100 Å pore size, Dionex, LC Packings) with a linear gradient of 5–80% acetonitrile in 0.1% formic acid. Sample was delivered over 120 min by a nano-LC ultra 1D plus system (Eksigent) at a 200 nL/min flow-rate to a stainless steel nano-bore emitter (OD 150 µm, ID 30 µm, Proxeon, Odense, Denmark). Peptides were scanned and fragmented with an LTQ XL linear ion trap mass spectrometer (Thermo, San Jose, CA) operated in data-dependent ZoomScan and MS/MS switching mode using the three most intense precursors detected in a survey scan from 400 to 1600 u (three µscans). ZoomScan mass window was set to 12 Da enabling monitoring of the entire ^12^C/^13^C isotopic envelope of most doubly and triply charged peptides. Singly charged ions were excluded for MS/MS analysis. Normalized collision energy was set to 35% and dynamic exclusion was applied during 3 min periods to avoid repetitive fragmentation ions.

#### Protein identification and quantitation

Generated .raw files were converted to .mgf files for MASCOT database search. A database containing the NCBInr Homo Sapiens sequences containing 34180 protein entries (as of 04-03-2008) was searched using MASCOT Software (version 2.3 Matrix Science) for protein identification. Search criteria included trypsin specificity with one missed cleavage allowed, and methionine oxidation, ^13^C-Arg and ^13^C-Lys as variable modifications. A minimum precursor fragment-ion mass accuracy of 1.2 and 0.3 Da, respectively, and a requirement of at least two bold red (unique peptides) per protein were required for protein quantitation. Cut-off values for MASCOT scores of peptides and proteins were set to 39 (*p*<0.05) and 46 (*p*<0.01), respectively, to consider them as accurate identifications. The false positive rate was calculated searching the same spectra against the NCBInr Homo Sapiens decoy randomized database. Relative quantification ratios of identified proteins were calculated using QuiXoT (version 1.3.26). SILAC T24T/T24 ratios were defined by the intensities of the heavy peptides (C^13^) divided by the intensities of the light peptides (C^12^). Protein ratios obtained by QuiXoT were manually verified for all peptides. A proportion of ^13^C_6_-Arg was converted to ^13^C_5_-Pro leading to a reduction in the intensity of the isotope-labeled peptide peak; this was corrected for all peptides containing one or more proline residues by adding the intensity found for the peptide containing ^13^C_6_-Arg ^13^C_5_-Pro or ^13^C_6_-Lys ^13^C_5_-Pro to the intensity of the peak containing only ^13^C_6_-Arg or ^13^C_6_-Lys. A combined list of proteins identified in all experiments was condensed at 80% homology using the ProteinCenter software package (Proxeon Bioinformatics AS, Odense, Denmark) to remove redundant IDs such as human orthologous sequences, redundant database entries, and indistinguishable isoforms based on observed peptide coverage. Subcellular localization and functional processes of the proteins identified by SILAC were assigned based on the biological knowledge available in Gene Ontology (GO) annotations. The Ingenuity Pathway (IPA) software was also used to provide insight into biological networks [33], [34].

### 3. Gene Expression Profiling with Oligonucleotide Arrays

#### RNA extraction

Total RNA was isolated using TRIzol (Life Technologies, Carlsbad, CA) followed by RNeasy purification. RNA quality was evaluated based on 260∶280 ratios of absorbance, and integrity was checked by gel electrophoresis using the 2100 Bioanalyzer (Agilent, Palo Alto, CA) [32], [34].

#### Gene arrays

Complementary DNA was synthesized by *in vitro* transcription from 1.5 µg of the total RNA purified using a T7-oligo(dT) Promoter Primer Assay (Affymetrix, Santa Clara, CA), labeled with biotinylated nucleotides (Enzo Biochem, Farmingdale, NY), and hybridized to test GeneChips (Affymetrix), to assess sample quality before hybridizing onto the U133A human GeneChips containing 22,283 probes representing known genes and expression sequence tags (Affymetrix) [34].

#### Data analysis

Scanned image files were visually inspected for artifacts and analyzed using the Affymetrix Microarray Suite 5.0 (MAS 5.0). Differential expression was evaluated using signal as the main response measure extracted for each gene in every sample, as determined by the default settings of the MAS 5.0. Correlations between gene and protein ratios were analyzed using Kendall’s tau test. To compare SILAC and oligonucleotide arrays results, the cumulative probability of expected and observed results were represented over the range of differential expression ratios.

### 4. Validation by immunoblotting

Total protein was extracted from bladder cancer cells using RIPA lysis buffer and quantified with the Bradford assay using BSA as standard (Protein Assay, Bio-Rad, Hercules, CA). Total protein extracts (50 µg) were mixed with 5x SDS sample buffer (62.5 mM TrisHCl [pH 6.8], 2% SDS, 10% glycerol, 5% β-mercaptoethanol, 0.005% bromophenol blue) and resolved by SDS-PAGE on 10% acrylamide gels. Proteins were electrotransferred onto PVDF membranes (Millipore, Bedford, MC) and activation with methanol. Membranes were blocked with 5% non-fat dry milk in PBS and 0.1% Tween-20 for 1 hour at room temperature and incubated overnight at 4 °C with primary antibodies against: Annexin2 (39 kDa, mouse, 1∶2000, #610068, BD Transduction Laboratories, San José, CA US), Bcas2 (26 kDa, mouse, 1∶6000, #H00010286-M01, Abnova, Heidelberg, Germany), L-Caldesmon (80kDa, mouse, 1∶100, #C56520, BD Transduction Laboratories), calreticulin (48 kDa, rabbit, 1∶5000, #C4606, Sigma, St. Louis, MO, US), Caveolin1 (20–22 kDa, mouse, 1∶100, #C37120, BD Transduction Laboratories), cdc2 (34 kDa, rabbit, 1∶1000, #sc-954, Santa Cruz, Santa Cruz, CA, US), CD44 (80 kDa, mouse, 1∶50, #NCL-CD44v3, Novocastra, Wetzlar, Germany), Copine3 (38 kDa, rabbit,1∶100, kindly supplied by Dr. Piris, located at CNIO, Madrid, Spain), Cul3 (89 kDa, rabbit, 1∶100, #RB1575PCS, NeoMarkers, Fremont, CA, US), Cytokeratin18 (48 kDa, mouse, 1∶100, #IF14, Oncogene-MERCK, Darmstad, Germany), DDX21 (87 kDa, rabbit,1∶3500, #10528-1-AP, Proteintech, US), DNMT1 (183 kDa, mouse, 1∶100, #IMG-261, IMGENEX, San Diego, CA, US), Dynactin p50 (44 kDa, mouse,1∶100, #D74620, BD Transduction Laboratories), Dynamin (mouse, 97 kDa, 1∶5000, #D25520, BD Transduction Laboratories,), EGFR (175 kDa, mouse, 1∶100, #GRO1, Oncogene-MERCK), Ezrin (80 kDa, mouse, 1∶7000, #E8897, Sigma), Filamin A (250 kDa, mouse, 1∶50, #NCL-FIL, Novocastra, UK), gelsolin (47 kDa, mouse,1∶100, #G4896, Sigma), HSP70 (70 kDa, mouse, 1∶200, #SC-66048, Santa Cruz), importin 9 (116 kDa, goat, 1∶100, sc-103567, Santa Cruz), MCM6 (92 kDa, rabbit, 1∶200, kindly supplied by Dr. Mendez, located at CNIO, Madrid, Spain), MAPK-4 (65 kDa, rabbit,1∶1000, sc-68169, Santa Cruz), Moesin (68–77 kDa, mouse,1∶50, #MS-727-P0, NeoMarkers), MSH6 (152 kDa, mouse,1∶200, #610918, BD transduction Laboratories), Nucleophosmin/B23 (32kDa, mouse, 1∶5000, #18-7288, Zymed, SF, CA, US), NUP133 (133 kDa, mouse,1∶500, #SC-101290, Santa Cruz), Rab14 (23 kDa, rabbit, 1∶100, #PRO-873, Avivasybio, San Diego, CA), RCC1 (44 kDa, goat, 1∶300, #SC-1161, Santa Cruz), VDAC (30 kDa, rabbit, 1∶100, #4866, Cell Signaling, Beverly, MA). Blots were washed in PBS and 0.1% Tween-20, and incubated with horseradish peroxidase-conjugated secondary antibodies for 1 h at room temperature: anti-mouse (1∶1000), anti-rabbit (1∶2000) and anti-goat (1∶2000, all Dako, Glostrup, Denmark). Antibody binding was visualised using an enhanced chemiluminescent immunoblotting detection system (ECL, GE Healthcare). α-tubulin (50kDa, mouse, 1∶4000, #T5168, Sigma) was used as loading and normalizing control. Immunoblots were scanned and analyzed using the ImageJ1.43u software (Wayne Rasband, National Institute of Health).

### 5. Clinical evaluation of the expression of metastases related biomarkers

#### Tissue samples and microarrays

Seven custom-made bladder cancer tissue microarrays were constructed at the Tumor Markers Group including triplicate or quadriplicate cores (1.0 mm) of primary bladder tumors (n = 284) following randomized designs. Paraffin-embedded tumors for tissue array construction were collected and handled anonymously following ethical and legal protection guidelines of human subjects after written consent approval and Institutional Review Board (IRB) approved protocols corresponding to the research project SAF2009-13035 at collaborating institutions: Fundacio Puigvert and Hospital Central de Asturias. Demographic information indicated the presence of 251 males and 33 females, with a median age of 66.0 years (range:25–81). Tumor stage distribution was: pT1 (n = 87), pT2 (n = 121), pT3 (n = 48) and pT4 (n = 28), and tumor grade distribution was: low-grade (n = 58) and high-grade (n = 226), defined according to consensus criteria [35]. Two of these tissue microarrays including a set of 71 muscle-invasive (pT2+) high grade TCC bladder tumors with known lymph node metastatic status (N0 = 37, N+ = 34). Clinicopathologic and annotated follow-up information allowed associations of Cul3 with histopathology and outcome.

#### Immunohistochemistry

Protein expression of Cul3 was assessed by immunohistochemistry on tissue microarrays using avidin-biotin immunoperoxidase procedures. Antigen retrieval (0.01% citric acid for 15 minutes under microwave) was employed prior to incubation overnight at 4 °C with the Cul3 rabbit antibody used in immunoblotting (1∶300 dilution). Antibody binding was detected with a biotinylated goat anti-rabbit secondary antibody (1∶1000, Vector Laboratories). Absence of primary antibody was used as negative control. Testis was utilized as positive control. Diaminobenzidine was utilized as the final chromogen and hematoxylin as the nuclear counterstain [32]–[34].

#### Statistical Analysis

Means of findings from two independent observers of all cores from each tumor sample arrayed were used for statistical analyses. Associations of Cul3 expression by immunohistochemistry with histopathologic stage and tumor grade were evaluated using the non-parametric Wilcoxon-Mann-Whitney and Kruskall-Wallis tests [36]. Cul3 expression was evaluated as a continuous variable based on the number of cells expressing the protein in the nucleus. The intensity of the staining was categorized as negative (−) to low (+), intermediate (++) and high (+++). In addition to the intracellular localization, it was also evaluated whether the protein was present or not in the extracellular matrix surrounding neoplastic cells. Cul3 cut-off level for prognostic evaluation was selected on the basis of median expression values among groups under analyses. Association of Cul3 with disease-specific survival was evaluated using the log-rank test in cases with available follow-up. Disease-specific survival time was defined as the months elapsed between transurethral resection or cystectomy and death as a result of disease (or the last follow-up date). Patients alive at the last follow-up or lost to follow-up were censored. Survival curves were plotted using Kaplan-Meier methodology [36]. Statistical analyses were performed using SPSS statistical package (version 17.0).

## Results

### Functional analyses *in vitro*

Several aggressiveness aspects of T24-T24T cells were initially analysed. T24T had significant higher proliferation rates than T24 at the four time points studied (p<0.05, Figure 1A). Invasion assays indicated that T24 were on average 50% less invasive than T24T cells at 48h (Figure 1B). Wound healing assays revealed significantly faster migration rate for T24T (Figure 1C). I*n vitro* assays suggested that T24T cells had more aggressive phenotypes.

### Changes in protein abundance between T24 and T24T cells using SILAC

A total of 1830 proteins were identified in the two SILAC experiments, from which 831 were simultaneously identified in both replicates and passed the criteria established for protein quantitation. The overall false discovery rate was 2.1% being estimated by the number of hits against the reverse sequence/total hits (p<0.01). The mean relative standard deviation (SD) of the ratios obtained from replicates was 0.24, indicating good agreement between experiments.

**Figure 2:**
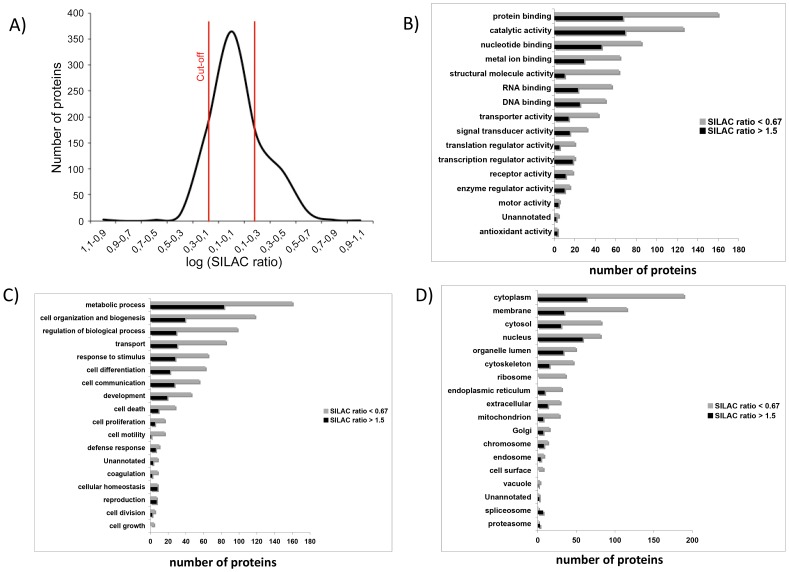
A) Distribution of SILAC T24T/T24 ratios: The log of the SILAC ratio for each protein (*n*  = 2) represents the difference in relative expression between highly metastatic (T24T) and invasive (T24) bladder cancer cells. Proteins were sorted and plotted by SILAC ratio. As expected for a 1∶1 mixture, most proteins showed a SILAC ratio within the 1.5 and 0.67 cutoffs. Classification of the proteins identified based on their functional annotations using the Gene Ontology: **B**) Molecular function, **C**) Biological processes and **D**) Cellular components. These analyses were performed with the 289 proteins found to be differentially expressed. When more than one assignment was available for a given protein, all the functional annotations were considered in the analyses. These classifications were redundant (over 100%) as proteins could be annotated in more than one assignment.

Regarding SILAC ratios distribution, most of the proteins identified were within the SILAC ratio range between 1.5 and 0.67, as expected when analysing closely related cell lines in a 1∶1 protein mixture (Figure 2A). Using 1.5 as the threshold ratio, 289 proteins were differentially expressed between the two cell lines, 88 of which were more abundant in T24T. Among the 289 differentially expressed proteins (Table S1), Table 1 includes those proteins previously related to bladder cancer metastases, and those validated by immunoblotting. The full list of proteins identified in both replicates (n = 831) using SILAC is in Table S2.

**Table 1:** Selected proteins with altered abundance in bladder cancer metastatic T24T versus T24 cells.

Accessionnumber(gi)	Protein Name	Commonname/Abbreviation	Molecularweight(Kda)	Gene ArrayT24T/T24Ratio	SILACT24T/T24Ratio	SD^1^
4507951	*tyrosine 3-monooxygenase/tryptophan 5-monooxygenase activation protein, eta polypeptide*	*YWHAH*	30	0.92	9.44	0.09
122939159	peptidyl arginine deiminase, type II	PADI2	75	–	8.35	0.69
41872631	*fatty acid synthase*	*FASN*	273	–	3.85	0.14
4504165	*gelsolin isoform a precursor*	GSN*	90	10.20	3.61	0.73
32171238	BAI1-associated protein 2-like 1	BAIAP2L1	56	–	3.03	0.05
4505591	*peroxiredoxin 1*	*PRDX1*	22	–	2.88	0.13
148298764	hydroxymethylglutaryl-CoA synthase 1	HMGCS1	57	1.17	2.83	0.25
38569421	*ATP citrate lyase isoform 1*	*ACLY*	120	10.74	2.54	0.17
10864011	sulfide dehydrogenase like	SQRDL	50	–	2.50	0.13
4507835	uridine monophosphate synthase	UMPS	52	1.18	2.34	0.20
4503165	*cullin 3*	Cul3*	89	1.00	2.26	0.18
4504169	*glutathione synthetase*	*GSS*	52	1.12	2.24	0.20
4503377	dihydropyrimidinase-like 2	DPYSL2	67	1.15	2.19	0.18
29789090	*regulator of chromosome condensation 2*	*RCC2*	56	–	2.19	0.28
20127454	5-aminoimidazole-4-carboxamide ribonucleotide formyltransferase/IMP cyclohydrolase	ATIC	64	–	2.15	0.20
21361709	regulation of nuclear pre-mRNA domain containing 1A	RPRD1A	35	–	2.09	0.25
47933397	lanosterol synthase	LSS	83	–	2.06	0.18
39777597	transglutaminase 2 isoform a	TGM2	77	1.04	2.01	0.04
116734860	*amylo-1, 6-glucosidase, 4-alpha-glucanotransferase isoform 1*	*AGL*	174	11.93	1.95	0.17
14150139	within bgcn homolog isoform 1	WIBG	22	–	1.94	0.06
20070384	phosphoglycerate mutase family member 5	PGAM5	32	–	1.92	0.18
4506903	*splicing factor, arginine/serine-rich 9*	*SFRS9*	25	1.04	1.9	0.05
gi|48255933	high-mobility group nucleosome binding domain 1	HMGN1	10	–	1.87	0.13
24308013	peptidase (mitochondrial processing) alpha	PMPCA	16	–	1.84	0.10
21361659	importin 9	IPO9*	116	1.09	1.83	0.17
29725609	**epidermal growth factor receptor isoform a precursor**	EGFR*	175	9.96	1.82	0.12
26051235	nucleoporin 133kDa	NUP133*	133	1.06	1.78	0.21
4507877	**vinculin isoform VCL**	**VCL**	123	0.09	0.67	0.03
48255935	**CD44 antigen isoform 1 precursor**	CD44*	80	0.83	0.66	0.06
4504047	**GNAS complex locus GNASL**	**GNAS**	45	1.03	0.61	0.08
161702986	*Ezrin*	EZR*	80	0.72	0.58	0.04
4504183	**glutathione transferase**	**GSTP1**	23	0.94	0.58	0.05
103472005	**antigen identified by monoclonal antibody Ki-67**	**MKI67**	358	–	0.56	0.05
4505257	*Moesin*	MSN*	68–77	0.89	0.54	0.04
55770844	**catenin, alpha 1**	**CTNNA1**	100	1.01	0.47	0.01
50845388	*annexin A2 isoform 1*	ANXA2*	39	0.97	0.42	0.04
4503015	copine III	CPNE3*	38	0.96	0.40	0.05
116063573	*filamin A, alpha isoform 1*	FLNA*	250	0.92	0.38	0.04
5031815	lysyl-tRNA synthetase isoform 2	KARS	68	1.04	0.31	0.03
156071459	*solute carrier family 25, member 5*	*SLC25A5*	35	0.98	0.30	0.06
19920317	cytoskeleton-associated protein 4	CKAP4	66	–	0.28	0.04
33620775	kinectin 1 isoform a	KTN1	14	1.03	0.26	0.04
209862851	plastin 3	PLS3	16	0.01	0.26	0.02
71773415	annexin VI isoform 2	ANXA6	75	0.90	0.24	0.01
105990514	filamin B, beta (actin binding protein 278)	FLNB	278	–	0.23	0.03
116805322	*gamma filamin isoform a*	*FLNC*	291	0.75	0.23	0.04
4507813	*UDP-glucose dehydrogenase*	*UGDH*	55	9.11	0.23	0.03
16753203	ubiquilin 1 isoform 1	UBQLN1	62	–	0.22	0.01
15451856	*caveolin 1*	CAV1*	20–22	0.96	0.21	0.04
7305053	myoferlin isoform a	MYOF	234	–	0.21	0.05
156104878	*Glutaminase*	*GLS*	73	1.05	0.20	0.03
42734430	polymerase I and transcript release factor	PTRF	43	–	0.20	0.03
157694492	MYB binding protein 1a isoform 2	MYBBP1A	133	1.12	0.20	0.14
63252913	*gelsolin-like capping protein*	*CAPG*	38	0.83	0.16	0.04
21071056	*SWI/SNF-related matrix-associated actin-dependent regulator of chromatin a4 isoform B*	*SMARCA4*	184	0.74	0.16	0.08
5453555	ras-related nuclear protein	RAN	24	–	0.07	0.11

All proteins were identified at >99% confidence (corresponding to a Mascot score >46). The table includes the accession number (gi), protein name, molecular weight (in kD), gene array ratio, SILAC ratios (T24T/T24), and the standard deviation (SD, n = 2). All proteins were identified in the two SILAC replicates with at least two unique peptides. Proteins previously described to be involved in cancer metastases are highlighted in italics, while those reported to be related to bladder cancer metastases are highlighted in bold. Proteins validated in immunoblots are highlighted with an asterisk. The absence of values in the "Gene Array Ratio" column, highlighted as "-", indicates absence of the specific probe on the array. The complete set of differentially expressed proteins identified is provided in Table S1.

### Functional classification of the proteins identified

The functional annotation of the 289 differentially expressed proteins in T24 and T24T cells was initially assigned using the Protein Center software. Three main types of annotations were obtained from GO consortium website: cellular components, molecular functions, and biological processes (Figure 2B, C, D). A GOslim approach defined specifically for ProteinCenter reduced the multiple GO annotations to a manageable set of approximately 20 high-level terms that were used to filter the information into percentage estimations. Major molecular functions included protein binding (78%) or catalytic activity (67%). Metabolic processes (84%) and cellular organization and biogenesis (54%) were frequent biological processes. Protein annotation distribution supported the *in vitro* functional assays described above linking cellular reorganization with migration and invasion phenotypes (Figure 1). A high number of proteins localized to the cytoplasm (87%) was found as compared to the nucleus (48%). This observation led us to focus on proteins that could play a relevant role in cytoskeletal reorganization and the aggressive phenotype of T24T.

### Comparison of gene and protein expression ratios

SILAC protein expression ratios were compared with mRNA expression provided by oligonucleotide microarrays for the candidates identified by both methods (n = 438) (Table 1, Table S3). A positive correlation coefficient (Kendalĺs tau) of 0.206 (p<0.0005) was obtained (Figure S1A). Importantly, the median SILAC protein expression ratio was 0.98 for these candidates (range: 0.16–9.44), which was similar to the median of 1.02 observed for oligonucleotide arrays (range: 0.01–100.80). Excluding two outliers detected by both techniques increased the correlation coefficient to 0.210 (p<0.0005, N = 438: Figure S1B). To interpret the differences between the expected and the observed correlations between RNA and protein expression, the cumulative probability of the observed ratio for differential expression was represented against the expected ratio for both techniques (Figure S1C, D). The figures highlighted the wider ranges of differential expression observed in oligonucleotide arrays when compared to the same candidates in SILAC analyses.

### Validation of SILAC identified candidates using immunoblotting

**Figure 3:**
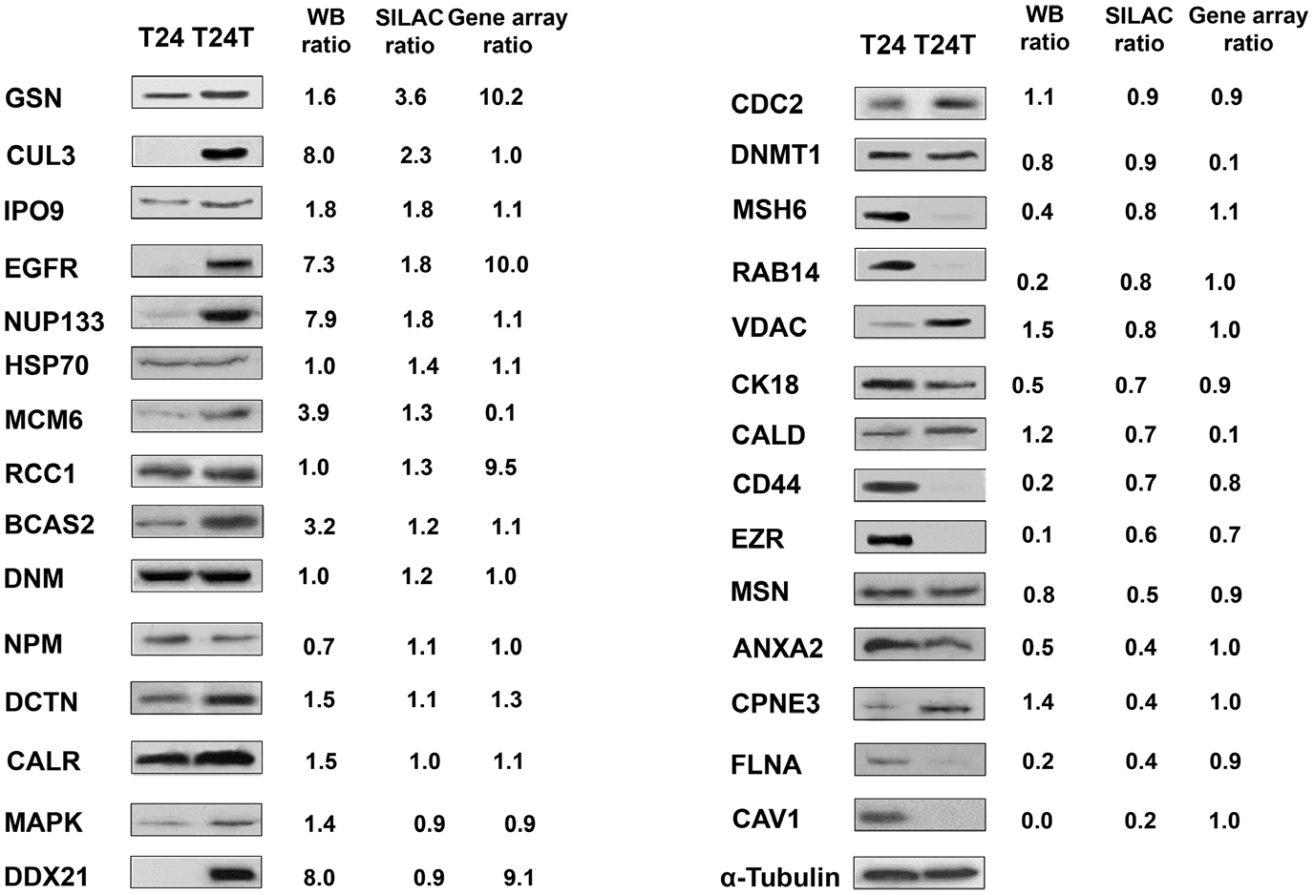
Verification of the expression of the proteins identified. (**A**) Validation of the SILAC results of selected proteins in immunoblots of protein extracts from the bladder cancer cells analyzed. The results validated the expression levels of proteins identified by the proteomic approach, including differentially and non-differentially expressed candidates. Antibodies displaying a single predominant band at the expected molecular weights were accepted: and α-tubulin, was used as the loading control. GSN, Gelsolin; Cul3, Cullin 3; IPO9. Importin 9; EGFR, Epidermal Growth Factor Receptor; NUP133, Nucleoporin 133; HSP70, Heat Shock Protein 70kDa; MCM6, Minichromosome Maintenance Complex Component 6; RCC1, Regulator of Chromosome Condensation 1; BCAS2, Breast Carcinoma Amplified Sequence 2; DNM, Dynamin; NPM, Nucleophosmin; DCTN, Dynactin; CALR, Calreticulin; MAPK, Mitogen-Activated Protein Kinase; DDX21, DEAD (Asp-Glu-Ala-Asp) box polypeptide 21; CDC2: Cell Division Cycle 2; DNMT1, DNA (cytosine-5)-Methyltransferase 1; MSH6, MutS Homolog 6; RAB14, GTPase Rab14; VDAC, Voltage-Dependent Anion Channel; CK18, Cytokeratin 18; CALD, Caldesmon; CD44, CD44 antigen isoform 1 precursor 2; EZR, Ezrin; MSN, Moesin; ANXA2, Annexin A2; CPNE3, Copine 3; FLNA, Filamin A; CAV1, Caveolin 1. Western Blots were scanned and analyzed using α-tubulin as normalizing control.

To validate SILAC expression ratios of proteins identified in both replicates, immunoblotting was performed (Figure 3). Increased expression in T24T was observed for gelsolin, Cul3, importins, nucleoporins and EGFR, and decreased expression was found for ezrin, moesin, filamin, caveolin or CD44, among others. Immunoblots were quantified to correlate with expression ratios obtained by SILAC and in gene arrays. Based on the good agreement of these observations for Cul3, a protein known to be involved in the ubiquitination and subsequent degradation of target proteins, it was selected for further analyses to: a) evaluate its clinical relevance as a biomarker candidate to assess aggressive clinical behaviour, and b) to evaluate Cul3 impact on the aggressive phenotype of T24T and on modulating expression of other differentially expressed proteins identified by SILAC. Figure S2 showed the additional validation by immunoblots of candidates differentially expressed in T24T cells in oligonucleotide arrays that were not quantified by SILAC, and *vice versa*, for which antibodies were available. We did not observe major differences in experimental molecular weights as compared to predicted sizes.

### Molecular pathways associated with aggressiveness

To understand the mechanisms by which differentially expressed proteins contribute to bladder cancer aggressiveness, the dataset containing the differentially expressed proteins (N = 289) was uploaded into the IPA software. An interaction map grouped 31 of the differentially expressed proteins to which Cul3 was added (Figure S3). An independent analysis was performed importing the top ten selected differentially expressed proteins in both SILAC and gene arrays, and validated in immunoblots (Figure S4). This analysis highlighted that validated proteins contributing to this network participated in the following critical neoplastic-related annotated biological functions: cellular assembly and organization, cancer, cell movement, cell morphology, and cell function and maintenance.

### Cul3 is differentially expressed in bladder tumors and associated with bladder cancer aggressiveness

**Figure 4:**
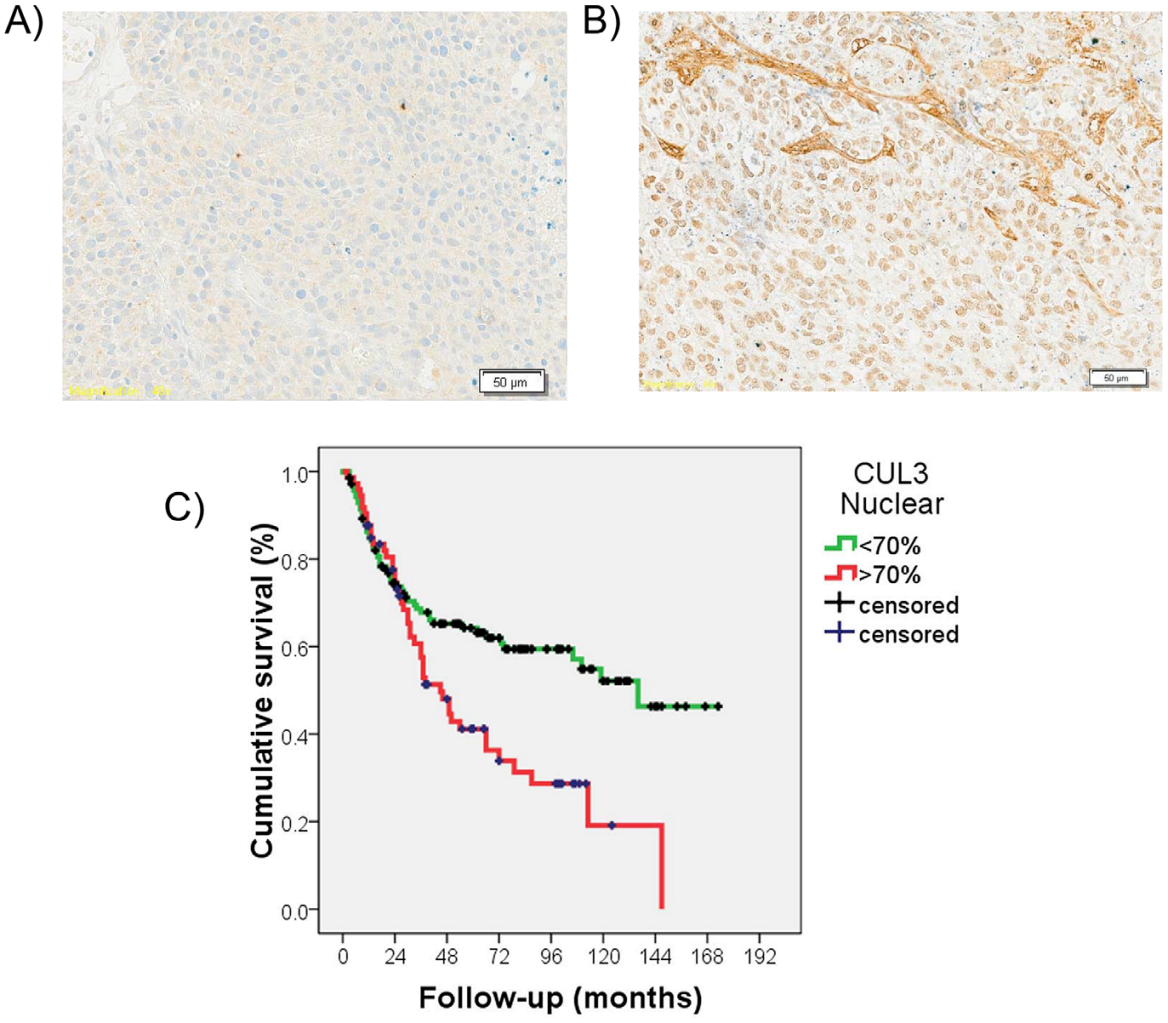
Clinical validation analyses of the differential expression of Cul3 in bladder cancer progression. (**A, B**) Representative immunohistochemistry expression patterns of Cul3 in non-invasive (A) and invasive (B) bladder tumors contained in tissue arrays. Strong expression of Cul3 was observed in invasive bladder tumors when compared to non-invasive lesions. Cul3 can also be observed in the extracellular matrix in B. There was a significant difference regarding the expression of Cul3 regarding tumor stage (p = 0.001: Original magnifications: x200). (**C**) Kaplan-Meier curve survival analysis indicating that increased nuclear Cul3 protein expression assessed by immunohistochemistry in tissue arrays was significantly associated with poor disease-specific survival (p = 0.002).

Protein expression patterns of Cul3 by immunohistochemistry were optimized and assessed on tissue arrays. Differential expression was observed for Cul3 among the bladder tumors tested. Significant statistical associations were found between Cul3 nuclear over-expression and increasing tumor stage when comparing non-invasive (Figure 4A) versus muscle-invasive (Figure 4B) bladder tumors (p = 0.001, n = 284). Moreover, Cul3 over-expression was associated with poor disease-specific survival (log-rank, p = 0.002), (Figure 4C). Primary invasive bladder tumors that developed lymph node metastases showed higher expression levels of Cul3 as compared to those with negative lymph nodes (p = 0.025). A high intensity and the presence of Cul3 in the extracellular matrix were also associated with increasing stage (p = 0.004, and p = 0.005, respectively), and with the presence of lymph node metastasis (p = 0.002, and p = 0.001). These observations indicated that Cul3 over-expression could be associated with tumor staging and the metastatic phenotype. Overall, expression patterns of Cul3 in bladder tumors suggested its role as a biomarker for tumor stratification, metastasis and clinical outcome prognosis.

### Functional and immunoblotting analyses upon Cul3 silencing

**Figure 5:**
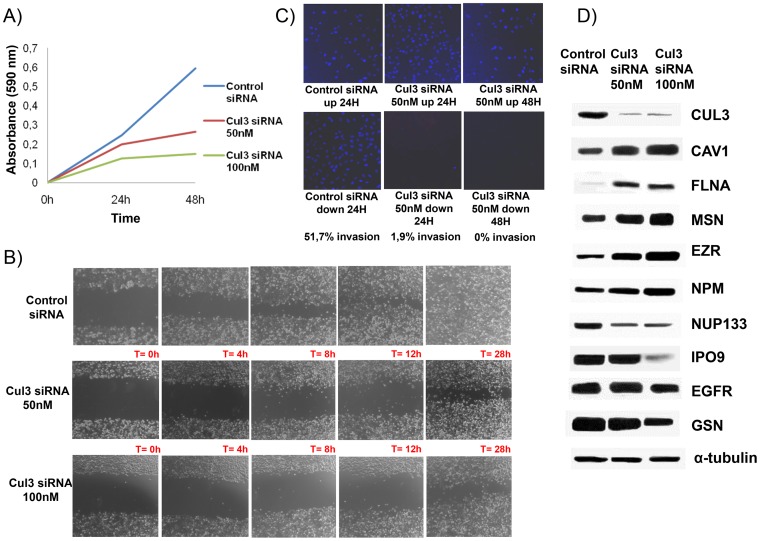
Functional analyses of the impact of Cul3 silencing on: A) proliferation, B) migration, and C) invasion. The average of duplicate experiments of each functional assay with siRNAs against Cul3 versus the control siRNA is represented in each panel. **D)** Immunoblotting of proteins found differentially expressed by SILAC upon Cul3 silencing on T24T cells. The results suggested that the differential expression of several of these proteins would be likely regulated by Cul3. Antibodies displaying a single predominant band at the expected molecular weights were accepted: Cul3, Cullin 3; CAV1, Caveolin 1; FLNA, Filamin A; MSN, Moesin; EZR, Ezrin; NPM, Nucleophosmin; NUP133, Nucleoporin 133; IPO9. Importin 9; EGFR, Epidermal Growth Factor Receptor; GSN, Gelsolin; and α-tubulin, was used as the loading control.

The impact of knocking down Cul3 expression using siRNA at 50nM and 100nM in the aggressive phenotype of T24T cells was assessed *in vitro*. Proliferation diminished at 24 and 48 hours after Cul3 silencing (p<0.05) (Figure 5A). Wound healing assays revealed the slower migration rate of T24T cells lacking Cul3 expression (Figure 5B). Invasion assays indicated that T24T cells silenced for Cul3 were on average 50% less invasive at both time points than the control siRNA (Figure 5C). Using Cul3 siRNAs at 100nM showed similar invasion rates as 50 nM (data not shown). The impact of Cul3 silencing on the expression of other proteins found differentially expressed by SILAC was tested by immunoblots (Figure 5D). Cul3 silencing restored the expression of cytoskeleton adhesion proteins such as filamin A, ezrin, caveolin1 or moesin. Overall, functional analyses and immunoblotting validation upon Cul3 silencing revealed that Cul3 modulated the aggressive phenotype of T24T, and modified the expression of cytoskeleton proteins also identified differentially expressed by SILAC.

## Discussion

A SILAC approach was designed to identify pathways associated with bladder cancer aggressiveness. Cul3 was revealed as a candidate contributing to the aggressive phenotype of T24T modifying cytoskeleton remodelling and as a bladder cancer biomarker correlating with poor outcome. Our comparative functional analyses of T24-T24T were complementary and agreed with previous *in vitro* results describing a more aggressive phenotype of T24T cells. By contrast to earlier analyses [5], we performed proliferation by seeding cells at a three-fold higher density, plus wound healing and invasion assays. These data highlighted the ability of T24T cells to grow on top of each other, in contrast to the contact inhibition previously described for T24 cells. These results further suggested that T24T cells have a greater potential for proliferation, motility and potentially to metastasize, as demonstrated *in vivo* [6]–[9]. A high number of proteins were found differentially expressed between T24-T24T, with biological network annotations supporting the functional differences observed *in vitro*. Furthermore, proteins were shown differentially expressed using oligonucleotide arrays and by selected immunoblotting. Immunostaining of tissue arrays containing independent series of bladder cancer patients served to assess the associations of a selected protein, Cul3, with clinicopathological variables. Functional analyses and immunoblotting validation upon Cul3 silencing highlighted its impact in the aggressive phenotype of T24T cells and at modulating other cytoskeleton proteins identified by SILAC. Thus, combination of -omic approaches, functional and clinical analyses identified Cul3 as a novel candidate related to bladder cancer aggressiveness.

The extent of the proteomic profile defined in this study was comparable to other SILAC studies [13]–[29]. On the basis of the identity and biological abundance of the proteins identified, SILAC exhibited a satisfactory dynamic range in profiling both high- and low-abundance proteins. The broad spectrum of proteins observed reflects SILAC suitability for proteomic studies of cancer cells. Subcellular fractionation reduced sample complexity and increased the probability of detecting less abundant proteins. The level of ambiguity for a protein ratio was estimated taking into account the SDs within each protein because every SILAC ratio was calculated as a mean of at least 2 peptide values with their associated SDs. We selected 1.5 and 0.67 as cutoffs, also frequently used in SILAC-related studies [13], [16], [23], [30]. When comparing two closely-related cell lines, it is expected that most of the proteins are expressed at similar levels. Indeed, most of the SILAC ratios were within the 0.67–1.5 range, apart from those related with the difference between these cells at their steady states (that could be attributed to their different phenotype). In SILAC, normalization was performed using the original mixture of the cells at a 1∶1 ratio and reaching 100% labeling efficiency for both cell populations.

The limitation of selecting a threshold of expression to consider proteins to be differentially expressed requires a follow-up validation analysis for key data. Verification of changes by two independent analytical methods, and using independent *in vitro* strategies and clinical material provided confidence that the experimental design permitted significant changes in abundance to be validated. The limited correlation between transcript and protein expression at their steady state was similar to the 0.28 previously reported in pancreatic cells [14]. This could be attributed to the wider range of ratios of expression measured by gene arrays while the majority of the SILAC ratios were in the low range. SILAC ratios were more limited due to the internal labelling and the characteristic 1∶1 mixture of the protein extracts analyzed. The weak correlation between the gene array and SILAC ratios highlighted the relevance of quantitative proteomic approaches to estimate the expression of proteins of interest (not always predictable based on transcript levels), in concordance with previous reports [14]. There were missing data between both techniques because not all the coding products of the genes measured by the early version of the Affymetrix oligonucleotide array (U133A) were detected by SILAC. Similarly, genes coding for the 831 proteins identified by SILAC duplicates were not included among the probes contained in the commercial U133 oligonucleotide array. Availability of both transcript and protein expression levels could also be utilized to uncover potential regulatory mechanisms modifying translation or protein degradation. Immunoblotting validation was closely correlated to the SILAC results, and also served to validate candidates identified in oligonucleotide arrays (Figure S2).

Cul3 was selected from the top over-expressed candidates in T24T not previously characterized in bladder cancer for which we had available reagents for further studies. Cul3 was differentially expressed in T24T using three different methodologies: SILAC, gene arrays and immunoblotting. Cul3 is one of the four members of the cullin protein family [37], [38]. It belongs to the core component of multiple ubiquitin-protein ligase complexes that mediate the ubiquitination and subsequent proteasomal degradation of their target proteins [39], [40]. Cul3 acts as a scaffolding protein in a heterodimeric complex playing a central role in the specificity of polyubiquitinization of these proteins, positioning the substrate and the ubiquitin-conjugating enzyme [38], [41]. Although the full list of targets whose ubiquitination and degradation is mediated by Cul3 remains unknown, cancer-related proteins reported include cyclin E [42], or Rho [43], among others [42]–[45]. In concordance with the interaction network shown in Figure S3, it could be proposed that Cul3 would be involved in the proteasomal degradation of adhesion associated cytoskeletal proteins such as filamin A, ezrin, caveolin1 or moesin. Indeed, the expression of these proteins increased upon Cul3 silencing, observations highlighting the impact of Cul3 expression not only on the aggressive phenotype of T24T shown by functional assays, but also modifying the expression of other proteins identified by SILAC. It remains to be characterized whether Cul3 might be directly involved in the proteasomal degradation of cytoskeleton proteins, potentially regulating the migration and invasive aggressiveness properties of T24T cells. Regarding therapeutic implications, members of the cullin family are covalently modified by NEDD8, where Cul3 ubiquitating ligase functioned as a NEDD8-bound heterodimer [46]. Neddylation and deneddylation may regulate Cul3 protein accumulation [47], suggesting new approaches to treat cancer by inhibiting the NEDD8-activated-cullin ligases [48]. To our knowledge, this is the first study evaluating Cul3 by immunohistochemistry, not only in bladder cancer but also in human tumors. Our findings were innovative and clinically relevant since Cul3 expression was linked to the invasive/metastatic phenotype in human bladder tumors, and also revealed that this protein can be secreted to the extracellular matrix. Our results highlighted the impact of the ubiquitin-proteasome pathway in bladder cancer aggressiveness, uncovering a novel biomarker and pathway potentially exploited therapeutically. Further focused designed studies are warranted to dissect the clinical relevance of Cul3 expression patterns in specific bladder cancer subgroups and address their specific clinical outcome endpoints.

The proteomic approach identified differential expression of proteins previously linked with aggressive clinical outcome in bladder tumors: gelsolin [49], moesin [32], Ezrin [50], caveolin [32], Filamin A [33]. The large number of differentially expressed proteins localized to the cytoplasm highlighted the relevance of adhesion molecules and cytoskeletal reorganization in bladder cancer aggressiveness (suported also by the IPA analysis), which could justify the higher proliferative, migration and invasive rate of T24T. Cul3 was uncovered as a clinically and biologically relevant candidate, which could promote cancer aggressiveness by regulating the expression of other critical cancer-related proteins [48]–[50]. Further research is warranted to define how cytoskeleton remodelling of these proteins specifically contribute to bladder cancer aggressiveness.

### Concluding Remarks

The SILAC approach served to identify potential candidates involved in bladder cancer aggressiveness in vitro. Functional and clinical validation analyses served to uncover the roles of Cul3 at regulating cytoskeleton remodelling, and as a progression and clinical outcome stratification biomarker.

## Supporting Information

Figure S1 **Comparison of the metastatic profile using gene profiling of a oligonucleotide array and SILAC. (A)** Dispersion plot of the ratios of expression (represented as circles) observed between the oligonucleotide arrays and SILAC considering the 438 candidates defined by both techniques. The outliers represent candidates with very high differential ratios by oligonucleotide arrays (around 100) and SILAC (around 10). **(B)** Dispersion plot of the ratios of expression (represented as circles) observed between the oligonucleotide arrays and SILAC, excluding the outliers with high expression in the oligonucleotide arrays (>100) and in the SILAC (>9). Even after excluding the outliers, while the range of expression of the ratios for oligonucleotide microarrays was extensive, in SILAC analyses the majority of the differential expression was mild in the low range of ratios. **(C)** Cumulative probabilities (represented as circles) of the observed differential expression ratio against the expected ratio for oligonucleotide arrays. **(A)** Cumulative probabilities (represented as circles) of the observed differential expression ratio against the expected ratio for SILAC approach.
(PPT)

Figure S2 **Western blotting validation of differentially expressed proteins in T24T when compared to T24 on the basis of the oligonucleotide arrays and that were not quantified using SILAC.** MMP2, Matrix Metalloproteinase 2; EphA1, Ephrin type-A receptor 1; MAGE 1, Melanoma associated antigen 1; IGFBP2, Insulin-like growth factor-binding protein 2; SOX9, Transcription factor SOX-9; PMF-1, Polyamine-modulated factor 1; SIVA, Apoptosis regulatory protein Siva; XRCC1, X-ray repair cross-complementing protein 1; ZYX, Zyxin; RAB6, Ras-related protein 6; MMP1, Matrix Metalloproteinase 1; CK2, Cytokeratin 2; FGFR1, Fibroblast growth factor receptor 1; CDK4, Cyclin-Dependent Kinase 4; REG1, Lithostathine 1; CLDN3, Claudin 3; SDC, Syndecan; KISS1, Metastasis-suppressor KiSS-1; SYP, Synaptophysin; SOX4, Transcription factor SOX-4; ANXA1, Annexin A1; GGT-1, Gamma-glutamyltranspeptidase 1; BDNF-1, Brain-derived neurotrophic factor; NUP62, Nucleoporin 62; GAL3, Galectin 3; GRB2, Growth factor receptor-bound protein 2; COX2, Cyclooxigenase2. The antibodies were raised against the following protein (and the dilutions used in immunoblots are shown): Annexin1 (38 kDa, mouse, 1∶2000, #610066, BD Transduction Laboratories), BDNF (14–27 kDa, mouse, 1∶50, #MAB248, R&D Systems, Minneapolis, MN, US), CDK4 (30 kDa, rabbit, 1∶500, #SC-260, Santa Cruz), Claudin-3 (22kDa, rabbit, 1∶1000, #18-7340, Zymed, Paisley, UK), Cox2 (70 kDa, mouse, 1∶500, #35-8200, Zymed), Cytokeratin 2 (66 kDa, mouse, 1∶100, #65177, Progen Biotechnik GmbH, Heidelberg), EphA1 (24 kDa, rabbit,1∶50, #34-3300, Zymed), FGF Receptor (110 kDa, mouse,1∶100, #13-3100, Zymed), Galectin-3 (18 kDa, rabbit, 1∶40, #18-0393, Zymed), GGT-1 (30–35 kDa, mouse, 1∶200, #H00002678-M01, clone 1F9, Abnova), GRB2 (25kDa, mouse, #610112, BD Transduction Laboratories) IGFBP-2 (35 kDa, mouse, 1∶200, #MAB674, R&D Systems), KISS1 (16 kDa, rabbit, 1∶50, #3590, Biovision, CA, USA), MAGE1 (46 kDa, mouse, 1∶100, #MA454, Abcam, Cambridge, UK), MMP1 (54 kDa, mouse, 1∶2000, #IM35, MERCK), MMP2 (64–72 kDa, mouse, 1∶100, #MAB9021, clone 101721, R&D Systems), NUP62 (62 kDa, mouse, 1∶100, #N43620, BD Transduction Laboratories), PMF-1 (23 kDa, mouse, 1∶100, #P24620, BD Transduction Laboratories,), RAB6 (25 kDa, rabbit, 1∶100, #SC-310, Santa Cruz), Reg1 (rabbit, 20 kDa, 1∶1000 dilution, kindly supplied by Dr. Iovanna, located at Inserm, Marseille, France), SOX9 (65 KDa, goat, 1∶250, #AF3075, R&D Systems,), SOX4 (40–46 kDa, mouse, 1∶500, #H00006659-A01, Abnova), Synaptophysin (38 kDa, rabbit, 1∶100, #18-0130, Zymed), Syndecan (90 kDa, rabbit, 1∶100, #36-2900, Zymed), SIVA (37,5 kDa, goat, 1∶1000, #HM1334, Hypromatrix, Worcester, MA), XRCC1 (70 kDa, mouse, 1∶50, #SC-56254, Santa Cruz), Zyxin (83 kDa, mouse, 1∶100, #Z45420, BD). Western Blots were scanned and analyzed using α-tubulin as normalizing loading control.
(PPT)

Figure S3 **Functional networks of the proteins identified: in silico protein interaction analysis.** Molecular network obtained using the IPA software selected from the networks of differentially expressed proteins identified as it contained the highest number of the proteins identified by SILAC (n = 31). Addition of Cul3, the validated candidate, to this molecular network served to generate an interaction map connecting the novel candidate with other proteins identified through their previously described biological interactions. In this network, genes or gene products are represented as nodes, and the biological relationship between two nodes is represented as an edge. All edges are supported by at least one publication from the information stored in the Ingenuity knowledge database.
(PPT)

Figure S4 **Functional networks of the proteins identified: in silico protein interaction analysis.** Biological interaction networking highlighted on the map of the top ten differentially expressed proteins in SILAC and oligonucleotide arrays, and validated in Western blots, including Cul3. Accession number and T24T/T24 ratio values for the proteins identified in Table 1 were imported into IPA software to generate different molecular networks. In this network, genes or gene products are represented as nodes, and the biological relationship between two nodes is represented as an edge. All edges are supported by at least one publication from the information stored in the Ingenuity knowledge database. The intensity of the node colour indicates the degree of over- (red) or under- (green) expression in T24T when compared to T24. The legend of the interaction network and the relationships between molecules is also provided.
(PPT)

Table S1 **Proteins with altered abundance in bladder cancer metastatic cells.** All proteins were identified at >99% confidence (corresponding to a Mascot score >46). The table includes accession number (gi), protein name, molecular weight (in kD), gene array ratio, SILAC ratios and the standard deviation (SD, n = 2). All proteins were identified in the two SILAC replicates with at least two unique peptides. Proteins previously described to be involved in cancer metastases are highlighted in italics, while those reported to be related to bladder cancer metastases are highlighted in bold.
(DOC)

Table S2 **Protein ID and quantification.** Proteins are listed alphabetically according to Protein Name. Proteins were identified according to the NCBI human databases (NCBI GI #s given for each ID). The table includes the corresponding UniProt and IPI accession numbers where available, Mascot scores corresponding to the highest scoring occurrence of a given protein or peptide, and GO annotations. T24T/T24 SILAC ratio = Intensity of the heavy peptide (C^13^)/Intensity of the light peptide (C^12^).
(XLS)

Table S3 **Detailed information of ratios obtained from the proteins identified by SILAC (831 proteins, first sheet) and those measured simultaneously by oligonucleotide arrays (438 proteins, second sheet), including probe identification and gene description of the oligonucleotide arrays.**
(XLS)
