# Supplementary figures and images for: A Quantitative Proteomic Analysis Uncovers the Relevance of CUL3 in Bladder Cancer Aggressiveness
Source: PLoS One. 2013 Jan 8;8(1):e53328. doi: 10.1371/journal.pone.0053328 (PMC3540081; doi:10.1371/journal.pone.0053328)

## Slide 1
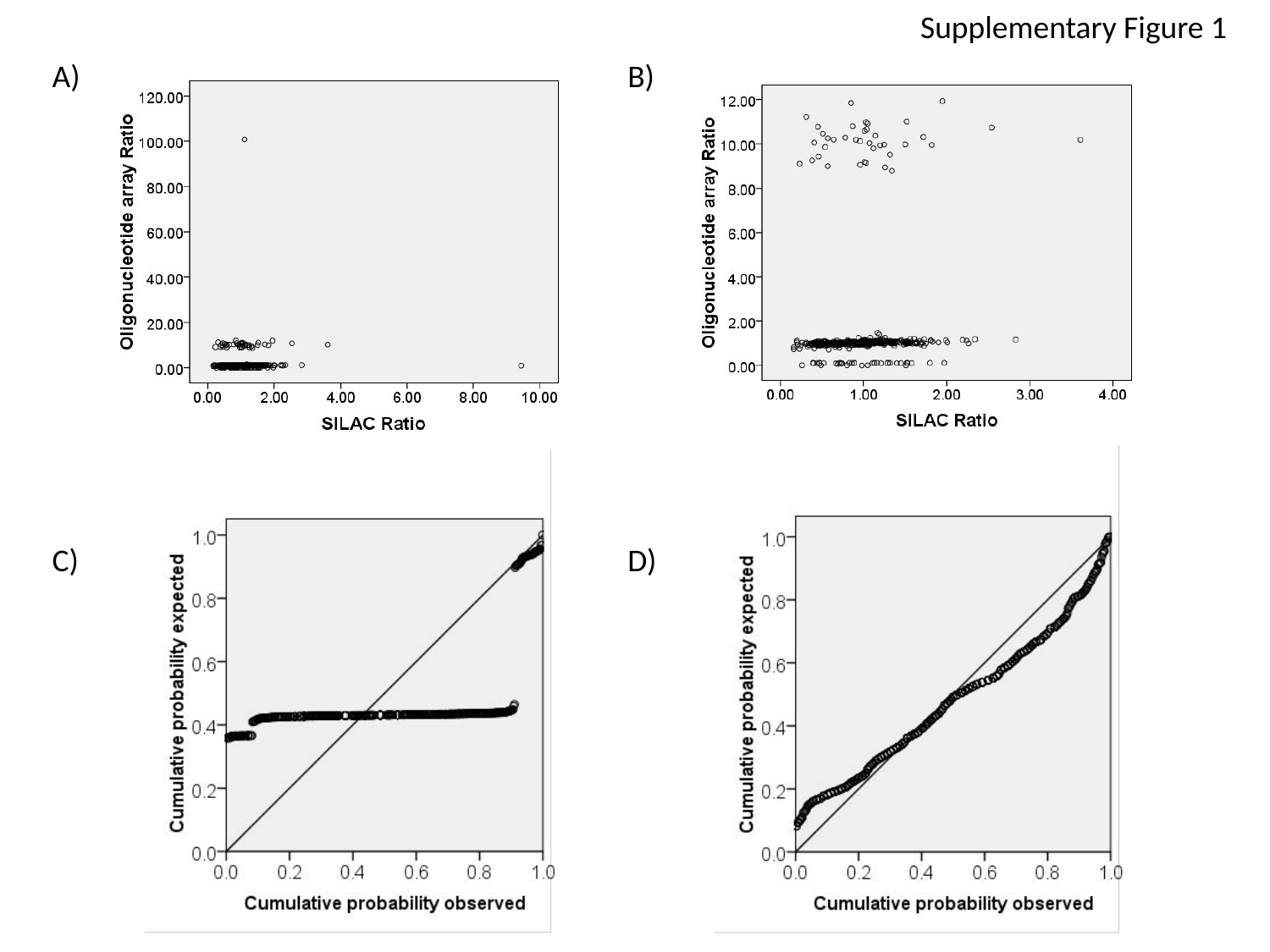

Supplementary Figure 1
A)
B)
C)
D)

Supplement: Figure S1 — Comparison of the metastatic profile using gene profiling of a oligonucleotide array and SILAC. (A) Dispersion plot of the ratios of expression (represented as circles) observed between the oligonucleotide arrays and SILAC considering the 438 candidates defined by both techniques. The outliers represent candidates with very high differential ratios by oligonucleotide arrays (around 100) and SILAC (around 10). (B) Dispersion plot of the ratios of expression (represented as circles) observed between the oligonucleotide arrays and SILAC, excluding the outliers with high expression in the oligonucleotide arrays (>100) and in the SILAC (>9). Even after excluding the outliers, while the range of expression of the ratios for oligonucleotide microarrays was extensive, in SILAC analyses the majority of the differential expression was mild in the low range of ratios. (C) Cumulative probabilities (represented as circles) of the observed differential expression ratio against the expected ratio for oligonucleotide arrays. (A) Cumulative probabilities (represented as circles) of the observed differential expression ratio against the expected ratio for SILAC approach. (PPT) [file pone.0053328.s001.ppt]

## Slide 1
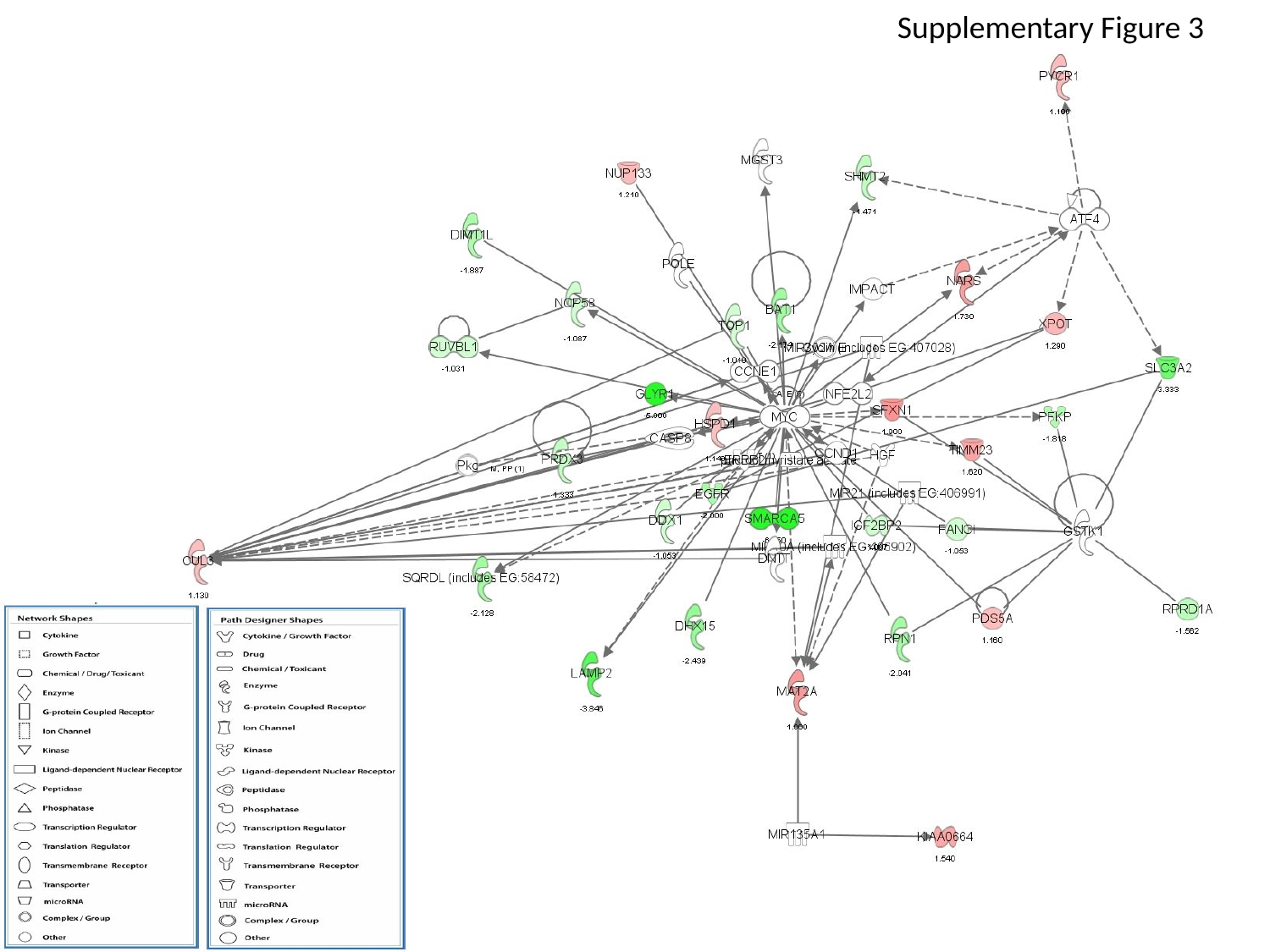

Supplementary Figure 3

Supplement: Figure S3 — Functional networks of the proteins identified: in silico protein interaction analysis. Molecular network obtained using the IPA software selected from the networks of differentially expressed proteins identified as it contained the highest number of the proteins identified by SILAC (n = 31). Addition of Cul3, the validated candidate, to this molecular network served to generate an interaction map connecting the novel candidate with other proteins identified through their previously described biological interactions. In this network, genes or gene products are represented as nodes, and the biological relationship between two nodes is represented as an edge. All edges are supported by at least one publication from the information stored in the Ingenuity knowledge database. (PPT) [file pone.0053328.s003.ppt]

## Slide 1
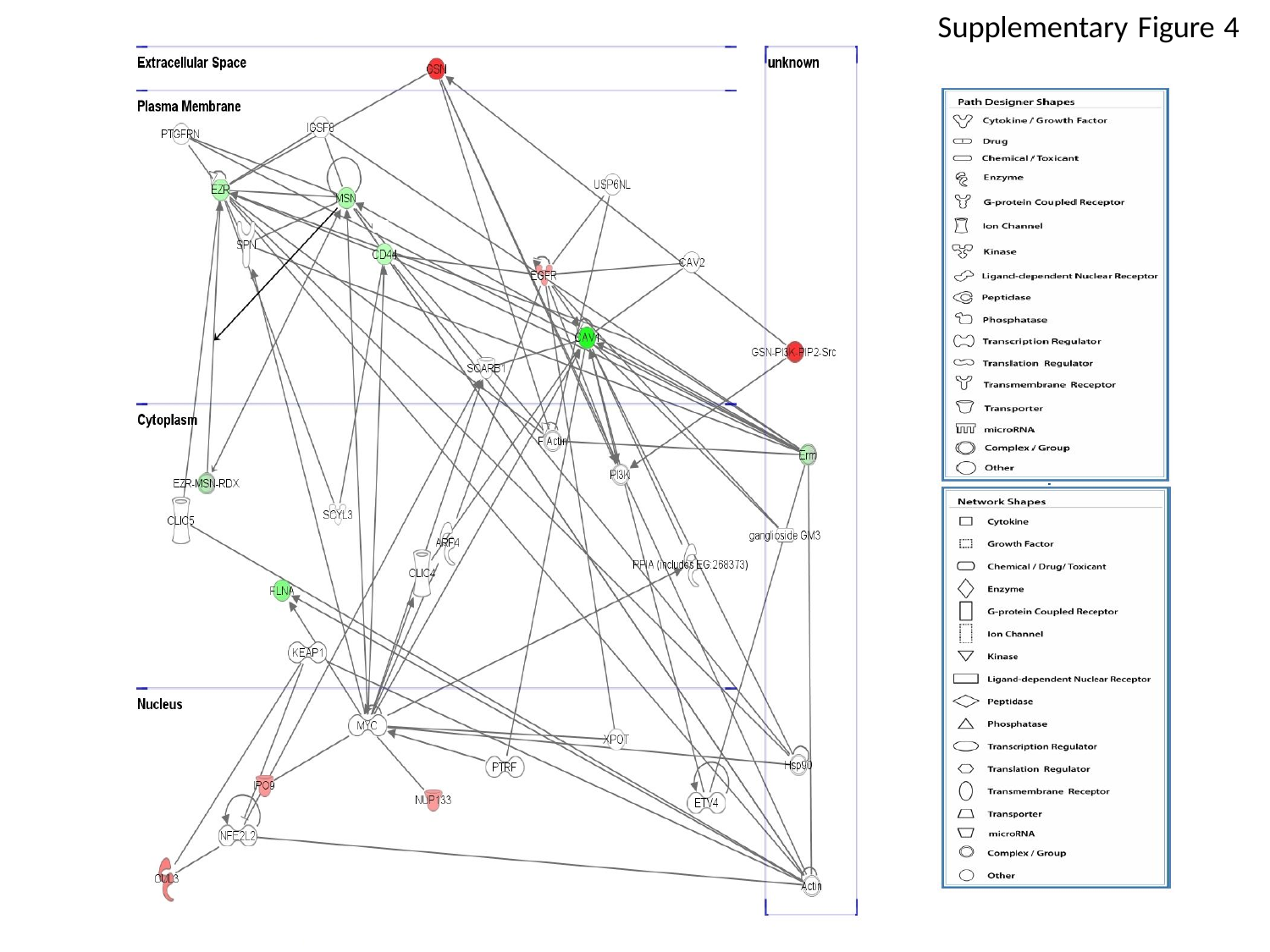

Supplement: Figure S4 — Functional networks of the proteins identified: in silico protein interaction analysis. Biological interaction networking highlighted on the map of the top ten differentially expressed proteins in SILAC and oligonucleotide arrays, and validated in Western blots, including Cul3. Accession number and T24T/T24 ratio values for the proteins identified in Table 1 were imported into IPA software to generate different molecular networks. In this network, genes or gene products are represented as nodes, and the biological relationship between two nodes is represented as an edge. All edges are supported by at least one publication from the information stored in the Ingenuity knowledge database. The intensity of the node colour indicates the degree of over- (red) or under- (green) expression in T24T when compared to T24. The legend of the interaction network and the relationships between molecules is also provided. (PPT) [file pone.0053328.s004.ppt]
