# Supplementary material for: A Quantitative Proteomic Analysis Uncovers the Relevance of CUL3 in Bladder Cancer Aggressiveness
Source: PLoS One. 2013 Jan 8;8(1):e53328. doi: 10.1371/journal.pone.0053328 (PMC3540081; doi:10.1371/journal.pone.0053328)
Supplement: Figure S2 — Western blotting validation of differentially expressed proteins in T24T when compared to T24 on the basis of the oligonucleotide arrays and that were not quantified using SILAC. MMP2, Matrix Metalloproteinase 2; EphA1, Ephrin type-A receptor 1; MAGE 1, Melanoma associated antigen 1; IGFBP2, Insulin-like growth factor-binding protein 2; SOX9, Transcription factor SOX-9; PMF-1, Polyamine-modulated factor 1; SIVA, Apoptosis regulatory protein Siva; XRCC1, X-ray repair cross-complementing protein 1; ZYX, Zyxin; RAB6, Ras-related protein 6; MMP1, Matrix Metalloproteinase 1; CK2, Cytokeratin 2; FGFR1, Fibroblast growth factor receptor 1; CDK4, Cyclin-Dependent Kinase 4; REG1, Lithostathine 1; CLDN3, Claudin 3; SDC, Syndecan; KISS1, Metastasis-suppressor KiSS-1; SYP, Synaptophysin; SOX4, Transcription factor SOX-4; ANXA1, Annexin A1; GGT-1, Gamma-glutamyltranspeptidase 1; BDNF-1, Brain-derived neurotrophic factor; NUP62, Nucleoporin 62; GAL3, Galectin 3; GRB2, Growth factor receptor-bound protein 2; COX2, Cyclooxigenase2. The antibodies were raised against the following protein (and the dilutions used in immunoblots are shown): Annexin1 (38 kDa, mouse, 1∶2000, #610066, BD Transduction Laboratories), BDNF (14–27 kDa, mouse, 1∶50, #MAB248, R&D Systems, Minneapolis, MN, US), CDK4 (30 kDa, rabbit, 1∶500, #SC-260, Santa Cruz), Claudin-3 (22kDa, rabbit, 1∶1000, #18-7340, Zymed, Paisley, UK), Cox2 (70 kDa, mouse, 1∶500, #35-8200, Zymed), Cytokeratin 2 (66 kDa, mouse, 1∶100, #65177, Progen Biotechnik GmbH, Heidelberg), EphA1 (24 kDa, rabbit,1∶50, #34-3300, Zymed), FGF Receptor (110 kDa, mouse,1∶100, #13-3100, Zymed), Galectin-3 (18 kDa, rabbit, 1∶40, #18-0393, Zymed), GGT-1 (30–35 kDa, mouse, 1∶200, #H00002678-M01, clone 1F9, Abnova), GRB2 (25kDa, mouse, #610112, BD Transduction Laboratories) IGFBP-2 (35 kDa, mouse, 1∶200, #MAB674, R&D Systems), KISS1 (16 kDa, rabbit, 1∶50, #3590, Biovision, CA, USA), MAGE1 (46 kDa, mouse, 1∶100, #MA454, Abcam, Cambridge, UK), MMP1 (54 kDa, mouse [file pone.0053328.s002.ppt]

## Slide 1
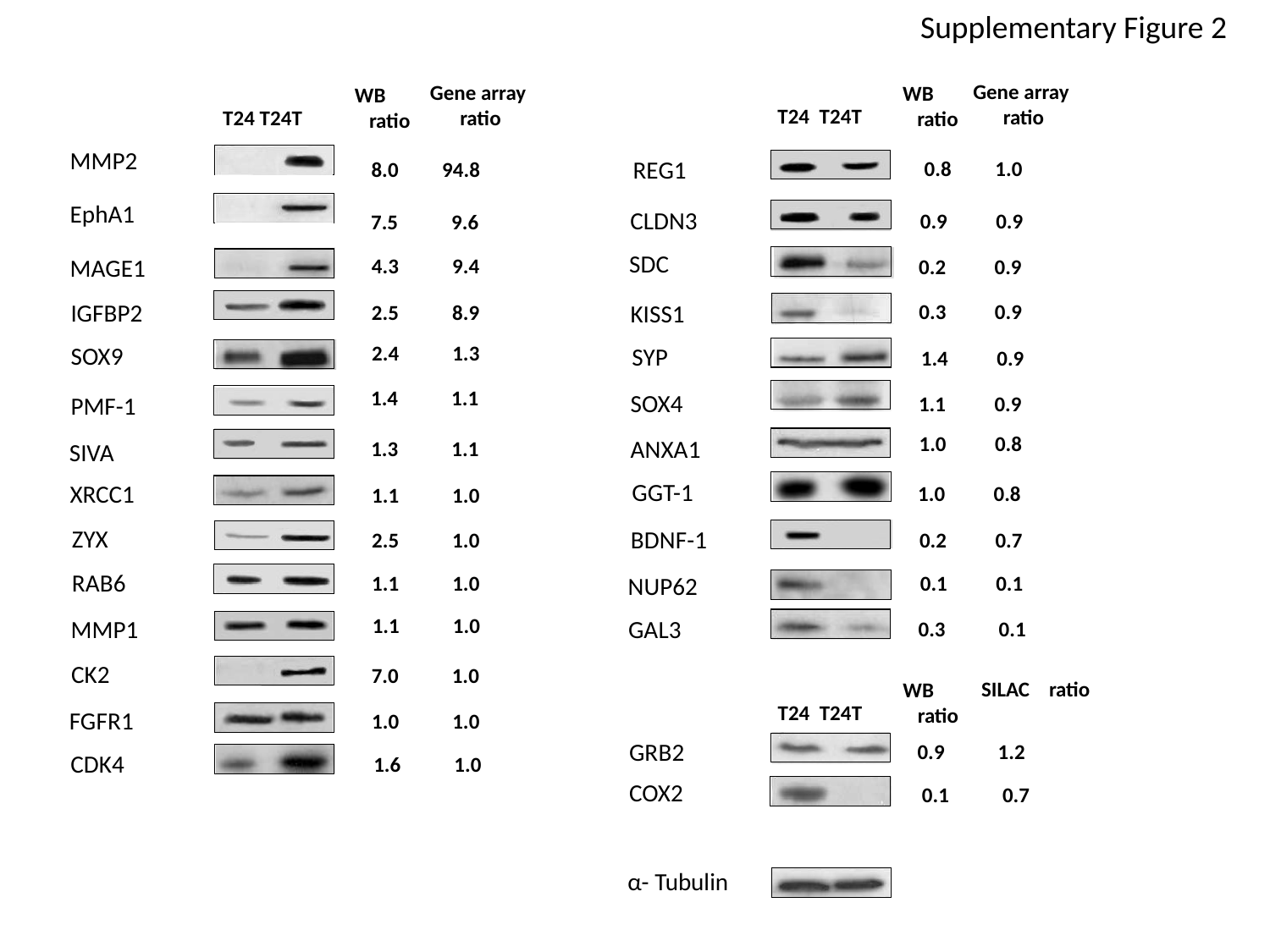

Supplementary Figure 2
Gene array ratio
Gene array ratio
WB ratio
WB ratio
T24 T24T
T24 T24T
MMP2
REG1
0.8 1.0
8.0 94.8
EphA1
CLDN3
0.9 0.9
7.5 9.6
SDC
MAGE1
4.3 9.4
0.2 0.9
IGFBP2
0.3 0.9
KISS1
2.5 8.9
2.4 1.3
SOX9
SYP
1.4 0.9
1.4 1.1
SOX4
PMF-1
1.1 0.9
1.0 0.8
ANXA1
1.3 1.1
SIVA
GGT-1
XRCC1
1.0 0.8
1.1 1.0
ZYX
BDNF-1
0.2 0.7
2.5 1.0
RAB6
1.1 1.0
NUP62
 0.1 0.1
1.1 1.0
MMP1
GAL3
0.3 0.1
CK2
7.0 1.0
SILAC ratio
WB ratio
T24 T24T
FGFR1
1.0 1.0
GRB2
0.9 1.2
CDK4
1.6 1.0
COX2
0.1 0.7
α- Tubulin
