# Supplementary material for: A Quantitative Proteomic Analysis Uncovers the Relevance of CUL3 in Bladder Cancer Aggressiveness
Source: PLoS One. 2013 Jan 8;8(1):e53328. doi: 10.1371/journal.pone.0053328 (PMC3540081; doi:10.1371/journal.pone.0053328)
Supplement: Table S1 — Proteins with altered abundance in bladder cancer metastatic cells. All proteins were identified at >99% confidence (corresponding to a Mascot score >46). The table includes accession number (gi), protein name, molecular weight (in kD), gene array ratio, SILAC ratios and the standard deviation (SD, n = 2). All proteins were identified in the two SILAC replicates with at least two unique peptides. Proteins previously described to be involved in cancer metastases are highlighted in italics, while those reported to be related to bladder cancer metastases are highlighted in bold. (DOC) [file pone.0053328.s005.doc]

*Supplementary Table 1*

| **Accession number ( gi|)** | **Protein Name** | **Common name/ Abbreviation** | **Molecular weight (Kda)** | **Gene Array Ratio** | **SILAC**  **Ratio** | **SD^1^** |
| --- | --- | --- | --- | --- | --- | --- |
| 4507951 | *tyrosine 3-monooxygenase/tryptophan 5-monooxygenase activation protein, eta polypeptide* | *YWHAH* | 30 | 0,92 | 9,44 | 0,09 |
| 122939159 | peptidyl arginine deiminase, type II | PADI2 | 75 | - | 8,35 | 0,69 |
| 41872631 | *fatty acid synthase* | *FASN* | 273 | - | 3,85 | 0,14 |
| 4504165 | *gelsolin isoform a precursor* | *GSN* | 90 | 10,20 | 3,61 | 0,73 |
| 32171238 | BAI1-associated protein 2-like 1 | BAIAP2L1 | 56 | - | 3,03 | 0,05 |
| 4505591 | *peroxiredoxin 1* | *PRDX1* | 22 | - | 2,88 | 0,13 |
| 148298764 | hydroxymethylglutaryl-CoA synthase 1 | HMGCS1 | 57 | 1,17 | 2,83 | 0,25 |
| 38569421 | *ATP citrate lyase isoform 1* | *ACLY* | 120 | 10,74 | 2,54 | 0,17 |
| 10864011 | sulfide dehydrogenase like | SQRDL | 50 | - | 2,50 | 0,13 |
| 4507835 | uridine monophosphate synthase | UMPS | 52 | 1,18 | 2,34 | 0,20 |
| 4503165 | *cullin 3* | *CUL3* | 89 | 1,00 | 2,26 | 0,18 |
| 4504169 | *glutathione synthetase* | *GSS* | 52 | 1,12 | 2,24 | 0,20 |
| 4503377 | dihydropyrimidinase-like 2 | DPYSL2 | 67 | 1,15 | 2,19 | 0,18 |
| 29789090 | *regulator of chromosome condensation 2* | *RCC2* | 56 | - | 2,19 | 0,28 |
| 20127454 | 5-aminoimidazole-4-carboxamide ribonucleotide formyltransferase/IMP cyclohydrolase | ATIC | 64 | - | 2,15 | 0,20 |
| 21361709 | regulation of nuclear pre-mRNA domain containing 1A | RPRD1A | 35 | - | 2,09 | 0,25 |
| 47933397 | lanosterol synthase | LSS | 83 | - | 2,06 | 0,18 |
| 39777597 | transglutaminase 2 isoform a | TGM2 | 77 | 1,04 | 2,01 | 0,04 |
| 122937211 | KIAA0368 protein | KIAA0368 | 204 | 1,13 | 1,99 | 0,17 |
| 31543419 | polynucleotide kinase 3'-phosphatase | PNKP | 57 | 0,11 | 1,97 | 0,11 |
| 116734860 | *amylo-1, 6-glucosidase, 4-alpha-glucanotransferase isoform 1* | *AGL* | 174 | 11,93 | 1,95 | 0,17 |
| 14150139 | within bgcn homolog isoform 1 | WIBG | 22 | - | 1,94 | 0,06 |
| 20070384 | phosphoglycerate mutase family member 5 | PGAM5 | 32 | - | 1,92 | 0,18 |
| 4506903 | *splicing factor, arginine/serine-rich 9* | *SFRS9* | 25 | 1,04 | 1,9 | 0,05 |
| gi|48255933 | high-mobility group nucleosome binding domain 1 | HMGN1 | 10 | - | 1,87 | 0,13 |
| 24308013 | peptidase (mitochondrial processing) alpha | PMPCA | 16 | - | 1,84 | 0,10 |
| 21361659 | importin 9 | IPO9 | 116 | 1,09 | 1,83 | 0,17 |
| 156564401 | N-ethylmaleimide-sensitive factor | NSF | 76 | 1,07 | 1,83 | 0,01 |
| 29725609 | **epidermal growth factor receptor isoform a precursor** | **EGFR** | 175 | 9,96 | 1,82 | 0,12 |
| 5174449 | H1 histone family, member X | H1FX | 22 | 1,14 | 1,81 | 0,14 |
| 209869995 | phosphoribosylglycinamide formyltransferase, phosphoribosylglycinamide synthetase, phosphoribosylaminoimidazole synthetase isoform 1 | GART | 107 | 1,11 | 1,81 | 0,01 |
| 4758762 | asparaginyl-tRNA synthetase | NARS | 63 | 0,11 | 1,80 | 0,01 |
| 20127486 | mannose 6 phosphate receptor binding protein 1 | M6PRBP1 | 47 | - | 1,80 | 0,06 |
| 5032179 | tripartite motif-containing 28 protein | TRIM28 | 57 | - | 1,80 | 0,19 |
| 83367072 | eukaryotic translation initiation factor 3, subunit 9 eta, 116kDa | EIF3B | 92 | - | 1,79 | 0,15 |
| 26051235 | nucleoporin 133kDa | NUP133 | 133 | 1,06 | 1,78 | 0,21 |
| 4504919 | *keratin 8* | *KRT8* | 41 | 1, 00 | 1,75 | 0,15 |
| 55770888 | early endosome antigen 1, 162kD | EEA1 | 162 | 0,88 | 1,73 | 0,14 |
| 92859701 | myosin VI | MYO6 | 149 | 1,18 | 1,73 | 0,07 |
| 24307939 | *chaperonin containing TCP1, subunit 5 (epsilon)* | *CCT5* | 59 | - | 1,72 | 0,11 |
| 31657129 | phosphoribosylformylglycinamidine synthase | PFAS | 80 | - | 1,72 | 0,15 |
| 11321607 | thyroid hormone receptor interactor 13 | TRIP13 | 45 | 10,31 | 1,72 | 0,04 |
| 5453607 | *chaperonin containing TCP1, subunit 7 isoform a* | *CCT7* | 55 | 1,04 | 1,71 | 0,06 |
| 55770906 | *XPA binding protein 2* | *XAB2* | 99 | - | 1,71 | 0,12 |
| 5803013 | *endoplasmic reticulum protein 29 isoform 1 precursor* | *ERP29* | 29 | - | 1,69 | 0,06 |
| 62460637 | importin 4 | IPO4 | 82 | 0,96 | 1,69 | 0,07 |
| 24797095 | pyrroline-5-carboxylate reductase 1 isoform 2 | PYCR1 | 33 | 1,02 | 1,69 | 0,14 |
| 4826952 | proteasome 26S non-ATPase subunit 5 | PSMD5 | 56 | - | 1,68 | 0,03 |
| 148491070 | *CTP synthase* | *CTPS* | 66 | 1,11 | 1,67 | 0,08 |
| 31543164 | WD repeat domain 58 isoform 1 | THOC6 | 37 | - | 1,66 | 0,03 |
| 4503729 | FK506 binding protein 52 | FKBP4 | 51 | 1,03 | 1,64 | 0,01 |
| 71037379 | glycogen phosphorylase, liver | PYGL | 97 | 1,06 | 1,64 | 0,15 |
| 5803191 | transcription elongation factor A 1 isoform 1 | TCEA1 | 34 | - | 1,64 | 0,02 |
| 38455427 | chaperonin containing TCP1, subunit 4 (delta) | CCT4 | 58 | 1,01 | 1,62 | 0,07 |
| 5031877 | *lamin B1* | *LMNB1* | 38 | 1,01 | 1,62 | 0,14 |
| 4506013 | protein phosphatase 1, regulatory subunit 7 | PPP1R7 | 41 | 1,22 | 1,61 | 0,11 |
| 15149476 | *arginyl-tRNA synthetase* | *RARS* | 23 | 0,99 | 1,59 | 0,06 |
| 31377800 | TROVE domain family, member 2 isoform 2 | TROVE2 | 59 | - | 1,59 | 0,11 |
| 148727247 | ubiquitin specific peptidase 5 isoform 2 | USP5 | 95 | 0,10 | 1,58 | 0,13 |
| 42741682 | zinc finger protein 265 isoform 2 | ZRANB2 | 37 |  | 1,58 | 0,14 |
| 48762932 | chaperonin containing TCP1, subunit 8 (theta) | CCT8 | 54 | 1,07 | 1,57 | 0,11 |
| 62240992 | *cysteinyl-tRNA synthetase isoform c* | *CARS* | 84 | 1,19 | 1,56 | 0,15 |
| 5032087 | splicing factor 3a, subunit 1, 120kDa isoform 1 | SF3A1 | 88 | 1,01 | 1,56 | 0,13 |
| 9910280 | UDP-glucose ceramide glucosyltransferase-like 1 isoform 1 | UGCGL1 | 177 | - | 1,56 | 0,04 |
| 115298668 | WD repeat domain 57 (U5 snRNP specific) | SNRNP40 | 39 | - | 1,56 | 0,09 |
| 5901922 | *cell division cycle 37 protein* | *CDC37* | 54 | 1,04 | 1,55 | 0,03 |
| 23510451 | N-acylaminoacyl-peptide hydrolase | APEH | 81 | 1,05 | 1,55 | 0,00 |
| 58331171 | chaperonin containing TCP1, subunit 6A isoform b | CCT6A | 7 | 1,05 | 1,54 | 0,09 |
| 4758112 | HLA-B associated transcript 1 | BAT1 | 40 | - | 1,54 | 0,05 |
| 5730027 | KH domain containing, RNA binding, signal transduction associated 1 | KHDRBS1 | 48 | - | 1,54 | 0,05 |
| 40217847 | activating signal cointegrator 1 complex subunit 3-like 1 | SNRNP200 | 216 | - | 1,53 | 0,11 |
| 5453603 | *chaperonin containing TCP1, subunit 2* | *CCT2* | 57 | 1,06 | 1,53 | 0,01 |
| 4503013 | copine I isoform a | CPNE1 | 59 | 1,04 | 1,53 | 0,20 |
| 87162455 | hypothetical protein LOC23277 | KIAA0664 | 146 | 0,11 | 1,53 | 0,11 |
| 117676403 | HIV-1 rev binding protein 2 | KRR1 | 43 | - | 1,52 | 0,01 |
| 4758844 | nucleoporin 155kDa isoform 2 | NUP155 | 155 | 0,11 | 1,52 | 0,06 |
| 21264343 | scaffold attachment factor B | SAFB | 107 | 11,02 | 1,52 | 0,25 |
| 4506891 | set | SET | 42 | 1,02 | 1,52 | 0,09 |
| 54112117 | splicing factor 3b, subunit 1 isoform 1 | SF3B1 | 87 | 1,01 | 1,52 | 0,13 |
| 57863257 | *T-complex protein 1 isoform a* | *TCP1* | 60 | 1,10 | 1,52 | 0,08 |
| 70166852 | adenosine deaminase, RNA-specific isoform a | ADAR | 136 | 1,03 | 1,51 | 0,05 |
| 4503971 | GDP dissociation inhibitor 1 | GDI1 | 50 | 0,97 | 1,51 | 0,07 |
| 4505749 | phosphofructokinase, muscle | PFKM | 81 | 1,08 | 1,51 | 0,03 |
| 4758304 | *protein disulfide isomerase-associated 4* | *PDIA4* | 73 | - | 1,51 | 0,03 |
| 4507135 | small nuclear ribonucleoprotein polypeptide N | SNRPN | 24 | 0,01 | 1,51 | 0,14 |
| 13654270 | LAS1-like | LAS1L | 49 | - | 1,50 | 0,18 |
| 7657671 | upstream binding transcription factor, RNA polymerase I isoform a | UBTF | 37 | 9,98 | 1,50 | 0,09 |
| 40254924 | leucine rich repeat containing 59 | LRRC59 | 34 | - | 0,67 | 0,04 |
| 28557677 | PHD finger protein 6 isoform 1 | PHF6 | 41 | - | 0,67 | 0,06 |
| 21361657 | protein disulfide-isomerase A3 precursor | PDIA3 | 56 | - | 0,67 | 0,03 |
| 24497620 | signal recognition particle 68kDa | SRP68 | 70 | - | 0,67 | 0,05 |
| 42518070 | tight junction protein 2 (zona occludens 2) isoform 1 | TJP2 | 133 | 0,96 | 0,67 | 0,01 |
| 4507669 | tumor protein, translationally-controlled 1 | TPT1 | 15 | 1,05 | 0,67 | 0,06 |
| 4507877 | **vinculin isoform VCL** | **VCL** | 123 | 0,09 | 0,67 | 0,03 |
| 12408656 | calpain 1, large subunit | CAPN1 | 81 | 1,02 | 0,66 | 0,03 |
| 48255935 | **CD44 antigen isoform 1 precursor** | **CD44** | 80 | 0,83 | 0,66 | 0,06 |
| 7705369 | coatomer protein complex, subunit beta 1 | COPB1 | 107 | 0,95 | 0,66 | 0,06 |
| 4557553 | emerin | EMD | 28 | - | 0,66 | 0,03 |
| 51477714 | mannosidase, alpha, class 2A, member 1 | MAN2A1 | 131 | 0,95 | 0,66 | 0,04 |
| 21389315 | solute carrier family 25 (mitochondrial carrier | SLC25A1 | 208 | - | 0,66 | 0,02 |
| 9910382 | mitochondrial import receptor Tom22 | TOMM22 | 15 | - | 0,65 | 0,03 |
| 5032093 | solute carrier family 1 member 5 isoform 1 | SLC1A5 | 34 | 1,04 | 0,65 | 0,03 |
| 23510340 | ubiquitin-activating enzyme E1 | UBA1 | 118 | - | 0,65 | 0,01 |
| 4757718 | actin-like 6A isoform 1 | ACTL6A | 47 | - | 0,64 | 0,01 |
| 14149734 | coronin, actin binding protein, 1B | CORO1B | 54 | - | 0,64 | 0,02 |
| 31542319 | epsilon subunit of coatomer protein complex isoform a | COPE | 34 | 10,20 | 0,64 | 0,05 |
| 4506787 | *IQ motif containing GTPase activating protein 1* | *IQGAP1* | 189 | 0,98 | 0,64 | 0,07 |
| 4505773 | *prohibitin* | *PHB* | 30 | 1,08 | 0,64 | 0,03 |
| 4506675 | ribophorin I precursor | RPN1 | 68 | 1,01 | 0,64 | 0,07 |
| 116256489 | septin 9 isoform c | SEPT9c | 65 | - | 0,64 | 0,05 |
| 21361497 | acyl-Coenzyme A dehydrogenase family, member 9 | ACAD9 | 68 | - | 0,63 | 0,07 |
| 4557469 | adaptor-related protein complex 2, beta 1 subunit isoform b | AP2B1 | 104 | 1,01 | 0,63 | 0,06 |
| 4758356 | *flap structure-specific endonuclease 1* | *FEN1* | 42 | 0,93 | 0,63 | 0,03 |
| 24431994 | unnamed protein product | CHID1 | 45 | - | 0,63 | 0,03 |
| 25188179 | voltage-dependent anion channel 3 isoform b | VDAC3 | 30 | - | 0,63 | 0,06 |
| 110611218 | ribosome binding protein 1 | RRBP1 | 41 | 0,97 | 0,62 | 0,10 |
| 24638454 | ATPase, Ca++ transporting, slow twitch 2 isoform 1 | ATP2A2 | 114 | - | 0,61 | 0,08 |
| 20070197 | dolichyl-diphosphooligosaccharide-protein glycosyltransferase precursor | DDOST | 50 | - | 0,61 | 0,06 |
| 4503475 | *eukaryotic translation elongation factor 1 alpha 2* | *EEF1A2* | 50 | 1,00 | 0,61 | 0,06 |
| 4504047 | **GNAS complex locus GNASL** | **GNAS** | 45 | 1,03 | 0,61 | 0,08 |
| 7661948 | malectin | MLEC | 32 | - | 0,61 | 0,04 |
| 41406064 | myosin, heavy polypeptide 10, non-muscle | MYH10 | 27 | 1,02 | 0,61 | 0,04 |
| 4503097 | casein kinase 2, alpha prime polypeptide | CSNK2A2 | 41 | 1,01 | 0,59 | 0,06 |
| 83281438 | eukaryotic translation initiation factor 3, subunit 1 alpha, 35kDa | EIF3J | 29 | - | 0,59 | 0,06 |
| 11055998 | guanine nucleotide-binding protein, beta-4 subunit | GNB4 | 37 | - | 0,59 | 0,03 |
| 7706751 | tubulin, gamma 2 | TUBG2 | 50 | 1,02 | 0,59 | 0,01 |
| 94721250 | vesicle-associated membrane protein-associated protein A isoform 1 | VAPA | 27 |  | 0,59 | 0,07 |
| 161702986 | *ezrin* | *EZR* | 80 | 0,72 | 0,58 | 0,04 |
| 4504183 | **glutathione transferase** | **GSTP1** | 23 | 0,94 | 0,58 | 0,05 |
| 21735415 | centromere protein B | CENPB | 65 | 1,02 | 0,57 | 0,06 |
| 7656991 | coronin, actin binding protein, 1C isoform 1 | CORO1C | 58 | 9,00 | 0,57 | 0,00 |
| 121114298 | cullin 4B isoform 1 | CUL4B | 103 | 1,05 | 0,57 | 0,05 |
| 29826335 | eukaryotic translation initiation factor 2 beta | EIF2S2 | 38 | - | 0,57 | 0,05 |
| 71361682 | nuclear mitotic apparatus protein 1 | NUMA1 | 238 | 10,26 | 0,57 | 0,07 |
| 4507357 | transgelin 2 | TAGLN2 | 22 | 0,96 | 0,57 | 0,03 |
| 8659555 | aconitase 1 | ACO1 | 98 | 0,93 | 0,56 | 0,00 |
| 103472005 | **antigen identified by monoclonal antibody Ki-67** | **MKI67** | 358 | - | 0,56 | 0,05 |
| 4501885 | *beta actin* | *ACTB* | 41 | 1,00 | 0,56 | 0,05 |
| 24797086 | importin 5 | IPO5 | 35 | 1,02 | 0,56 | 0,05 |
| 148747351 | protein kinase C and casein kinase substrate in neurons 2 | PACSIN2 | 55 | 1,03 | 0,56 | 0,03 |
| 35493916 | *ribophorin II isoform 1 precursor* | *RPN2* | 69 | - | 0,56 | 0,02 |
| gi|23618867 | sideroflexin 1 | SFXN1 | 35 |  | 0,56 | 0,06 |
| 87196351 | *DEAD/H (Asp-Glu-Ala-Asp/His) box polypeptide 3* | *DDX3X* | 73 | - | 0,55 | 0,03 |
| 11496885 | PDZ and LIM domain 7 isoform 1 | PDLIM7 | 49 | - | 0,55 | 0,05 |
| 5031703 | Ras-GTPase-activating protein SH3-domain-binding protein | G3BP1 | 52 | 0,95 | 0,55 | 0,05 |
| 4506725 | ribosomal protein S4, X-linked X isoform | RPS4X | 27 | 0,89 | 0,55 | 0,05 |
| 149363638 | septin 8 isoform c | SEPT8c | 55 | - | 0,55 | 0,08 |
| 66346679 | *SERPINE1 mRNA binding protein 1 isoform 1* | *SERBP1* | 49 | - | 0,55 | 0,08 |
| 39652628 | hypothetical protein LOC23196 | FAM120A | 121 | - | 0,54 | 0,08 |
| 116295258 | *integrin alpha 2 precursor* | *ITGA2* | 129 | 0,97 | 0,54 | 0,01 |
| 112380628 | *lysosomal-associated membrane protein 1* | *LAMP1* | 44 | 0,89 | 0,54 | 0,07 |
| 4505257 | *moesin* | *MSN* | 68-77 | 0,89 | 0,54 | 0,04 |
| 33286418 | pyruvate kinase, muscle isoform M2 | PKM2 | 57 | 9,86 | 0,54 | 0,01 |
| 24431933 | reticulon 4 isoform B | RTN4 | 129 | - | 0,54 | 0,06 |
| 24119203 | *tropomyosin 3 isoform 2* | *TPM3* | 18 | - | 0,54 | 0,03 |
| 4507903 | vaccinia related kinase 1 | VRK1 | 45 | 1,10 | 0,54 | 0,01 |
| 14210504 | adaptor-related protein complex 1, mu 1 subunit isoform 2 | AP1M1 | 48 | - | 0,53 | 0,03 |
| 4557317 | annexin A11 | ANXA11 | 54 | 1,10 | 0,53 | 0,04 |
| 67782362 | DEAH (Asp-Glu-Ala-His) box polypeptide 29 | DHX29 | 155 | - | 0,53 | 0,03 |
| 20149621 | dihydroxyacetone kinase 2 | DAK | 24 | - | 0,53 | 0,01 |
| 41322910 | *plectin 1 isoform 7* | *PLEC1* | 531 | 1,00 | 0,53 | 0,05 |
| 31621303 | sideroflexin 3 | SFXN3 | 35 | - | 0,53 | 0,06 |
| 21361103 | solute carrier family 25 (mitochondrial carrier, Aralar), member 12 | SLC25A12 | 74 | 1,07 | 0,53 | 0,06 |
| 156627575 | UDP-N-acteylglucosamine pyrophosphorylase 1 | UAP1 | 58 | - | 0,53 | 0,05 |
| 4506609 | ribosomal protein L19 | RPL19 | 23 | 0,10 | 0,52 | 0,06 |
| 4506741 | ribosomal protein S7 | RPS7 | 27 |  | 0,52 | 0,05 |
| 7661910 | tetratricopeptide repeat domain 35 | TTC35 | 34 | - | 0,52 | 0,03 |
| 47519616 | *tropomyosin 2 (beta) isoform 2* | *TPM2* | 32 | 1,09 | 0,52 | 0,02 |
| 30240932 | EH-domain containing 1 | EHD1 | 60 | 0,98 | 0,51 | 0,01 |
| 4885409 | high density lipoprotein binding protein | HDLBP | 100 | 10,47 | 0,51 | 0,06 |
| 4504897 | *karyopherin alpha 2* | *LOC728860 karyopherin* | 57 | - | 0,51 | 0,03 |
| 21361368 | pyrroline-5-carboxylate synthetase isoform 1 | ALDH18A1 | 87 | - | 0,51 | 0,01 |
| 4506661 | *ribosomal protein L7a* | *RPL7A* | 29 | 0,97 | 0,51 | 0,01 |
| 94429050 | SEC22 vesicle trafficking protein homolog B | SEC22B | 24 | - | 0,51 | 0,04 |
| 9966805 | DEAD (Asp-Glu-Ala-Asp) box polypeptide 24 | DDX24 | 96 | 1,05 | 0,50 | 0,05 |
| 4503481 | eukaryotic translation elongation factor 1 gamma | EEF1G | 50 | 1,02 | 0,50 | 0,05 |
| 4503515 | eukaryotic translation initiation factor 3, subunit 3 gamma, 40kDa | EIF3H | 39 | - | 0,50 | 0,05 |
| 4503519 | *eukaryotic translation initiation factor 3, subunit 5 epsilon, 47kDa* | *EIF3F* | 37 | - | 0,50 | 0,05 |
| 4503525 | eukaryotic translation initiation factor 3, subunit C | EIF3CL | 105 | - | 0,50 | 0,04 |
| 8923427 | *OCIA domain containing 1 isoform 1* | *OCIAD1* | 27 | - | 0,50 | 0,01 |
| 4506607 | ribosomal protein L18 | RPL18 | 21 | 1,04 | 0,50 | 0,01 |
| 148491091 | solute carrier family 25 member 24 isoform 1 | SLC25A24 | 25 | - | 0,50 | 0,05 |
| 31542947 | chaperonin | HSPD1 | 61 | 0,01 | 0,49 | 0,05 |
| 4758256 | eukaryotic translation initiation factor 2, subunit 1 alpha, 35kDa | EIF2S1 | 36 | 0,98 | 0,49 | 0,04 |
| 4504957 | *lysosomal-associated membrane protein 2 isoform A precursor* | *LAMP2* | 44 | 0,97 | 0,49 | 0,03 |
| 18104948 | ribosomal protein L21 | RPL21P16 | 22 | - | 0,49 | 0,03 |
| 14591909 | ribosomal protein L5 | RPL5 | 12 | 0,99 | 0,49 | 0,05 |
| 4506663 | ribosomal protein L8 | RPL8 | 28 | 1,02 | 0,49 | 0,04 |
| 61744475 | solute carrier family 3 (activators of dibasic and neutral amino acid transport), member 2 isoform a | SLC3A2 | 53 | 0,94 | 0,49 | 0,05 |
| 47419916 | tryptophanyl-tRNA synthetase isoform a | WARS | 53 | 0,96 | 0,49 | 0,05 |
| 4501881 | actin, alpha 1, skeletal muscle | ACTA1 | 42 | 0,88 | 0,48 | 0,06 |
| 21264315 | EH-domain containing 4 | EHD4 | 61 | - | 0,48 | 0,02 |
| 49472822 | eukaryotic translation initiation factor 3, subunit 4 delta, 44kDa | EIF3G | 35 | - | 0,48 | 0,04 |
| 21735596 | *programmed cell death 4 isoform 1* | *PDCD4* | 71 | 0,91 | 0,48 | 0,05 |
| 4506649 | ribosomal protein L3 isoform a | RPL3 | 33 | 1,02 | 0,48 | 0,02 |
| 17105394 | *similar to ribosomal protein L23A* | *hCG_16001* | 17 | - | 0,48 | 0,00 |
| 45439306 | aspartyl-tRNA synthetase | DARS | 73 | 1,09 | 0,47 | 0,01 |
| 55770844 | **catenin, alpha 1** | **CTNNA1** | 100 | 1,01 | 0,47 | 0,01 |
| 9910242 | GK001 | CCDC47 | 55 | - | 0,47 | 0,06 |
| 94721239 | isoleucyl-tRNA synthetase | IARS | 120 | 0,11 | 0,47 | 0,07 |
| 15431295 | ribosomal protein L13 | RPL13 | 24 | - | 0,47 | 0,01 |
| 16579885 | ribosomal protein L4 | RPL4 | 47 | 1,00 | 0,47 | 0,02 |
| 15431301 | ribosomal protein L7 | RPL7 | 29 | 0,93 | 0,47 | 0,03 |
| 15055539 | *ribosomal protein S2* | *RPS2* | 10 | 0,10 | 0,47 | 0,03 |
| 7661908 | Signal peptidase complex subunit 2 homolog (S. cerevisiae) | SPCS2 | 25 | - | 0,47 | 0,03 |
| 154759259 | spectrin, alpha, non-erythrocytic 1 (alpha-fodrin) isoform 2 | SPTAN1 | 284 | - | 0,47 | 0,06 |
| 112382250 | spectrin, beta, non-erythrocytic 1 isoform 1 | SPTBN1 | 274 | 0,88 | 0,47 | 0,06 |
| 4507237 | SSR alpha subunit | SSR1 | 29 | 1,03 | 0,47 | 0,06 |
| 5454122 | translocase of inner mitochondrial membrane 23 (yeast) homolog | TIMM23 | 39 | - | 0,47 | 0,00 |
| 30795231 | brain abundant, membrane attached signal protein 1 | BASP1 | 22 | 0,89 | 0,46 | 0,03 |
| 131888105 | cancer susceptibility candidate 1 isoform a | CASC1 | 83 | - | 0,46 | 0,07 |
| 119601264 | methylenetetrahydrofolate dehydrogenase (NADP+ dependent) 1, methenyltetrahydrofolate cyclohydrolase, formyltetrahydrofolate synthetase, isoform CRA_a | MTHFD1 | 101 | 9,44 | 0,46 | 0,01 |
| 15431288 | ribosomal protein L10a | RPL10A | 24 | 1,01 | 0,46 | 0,04 |
| 77404397 | *staphylococcal nuclease domain containing 1* | *SND1* | 101 | - | 0,46 | 0,05 |
| 98986464 | transmembrane emp24 domain-containing protein 10 precursor | TMED10 | 24 | - | 0,46 | 0,05 |
| 4503513 | eukaryotic translation initiation factor 3, subunit 2 beta, 36kDa | EIF3I | 36 | - | 0,45 | 0,03 |
| 4503521 | *eukaryotic translation initiation factor 3, subunit 6 48kDa* | *EIF3E* | 52 | - | 0,45 | 0,05 |
| 62241042 | glutamyl-prolyl tRNA synthetase | EPRS | 109 | 1,09 | 0,45 | 0,06 |
| 5174447 | *guanine nucleotide binding protein (G protein), beta polypeptide 2-like 1* | *GNB2L1* | 35 | 0,98 | 0,45 | 0,04 |
| 4506597 | ribosomal protein L12 | RPL12 | 17 | 1,01 | 0,45 | 0,05 |
| 4506617 | ribosomal protein L17 | LOC100133931 | 20 | - | 0,45 | 0,04 |
| 4506667 | ribosomal protein P0 | RPLP0 | 27 | 1,02 | 0,45 | 0,01 |
| 14141193 | ribosomal protein S9 | RPS9 | 13 | 10,78 | 0,45 | 0,02 |
| 19482174 | cullin 2 | CUL2 | 86 | 0,90 | 0,44 | 0,00 |
| 108773793 | *glucose-6-phosphate dehydrogenase isoform b* | *G6PD* | 59 | 1,04 | 0,44 | 0,03 |
| 124028525 | phenylalanyl-tRNA synthetase, beta subunit | FARSB | 65 | - | 0,44 | 0,07 |
| 27480190 | PREDICTED: similar to ribosomal protein L18a isoform 1 | LOC285053 | 20 | - | 0,44 | 0,02 |
| 15431293 | ribosomal protein L15 | RPL15 | 24 | 0,10 | 0,44 | 0,05 |
| 4506619 | ribosomal protein L24 | RPL24 | 17 | 1,03 | 0,44 | 0,01 |
| 16753227 | ribosomal protein L6 | RPL6 | 32 | 0,96 | 0,44 | 0,04 |
| 4506743 | ribosomal protein S8 | RPS8 | 24 | 1,02 | 0,44 | 0,04 |
| 50845388 | *annexin A2 isoform 1* | *ANXA2* | 39 | 0,97 | 0,42 | 0,04 |
| 4504301 | histone cluster 2, H4b | HIST2H4B | 11 | - | 0,42 | 0,05 |
| 14043022 | methionyl-tRNA synthetase | MARS | 101 | 1,06 | 0,42 | 0,00 |
| 12667788 | *myosin, heavy polypeptide 9, non-muscle* | *MYH9* | 226 | - | 0,42 | 0,05 |
| 6912634 | ribosomal protein L13a | *RPL13A* | 16 | 1,03 | 0,42 | 0,02 |
| 4506629 | *ribosomal protein L29* | *RPL29* | 17 | 1,03 | 0,42 | 0,05 |
| 13129092 | transmembrane protein 109 | TMEM109 | 26 | - | 0,42 | 0,00 |
| 4758012 | *clathrin heavy chain 1* | *CLTC clathrin* | 191 | 1,00 | 0,41 | 0,04 |
| 34098946 | nuclease sensitive element binding protein 1 | YBX1 | 29 | - | 0,41 | 0,03 |
| 46367787 | *poly(A) binding protein, cytoplasmic 1* | *PABPC1* | 29 | - | 0,41 | 0,01 |
| 4506749 | *ribonucleoside-diphosphate reductase M1 chain* | *RRM1* | 90 | 1,02 | 0,41 | 0,04 |
| 4506723 | ribosomal protein S3a | RPS3A | 29 | 0,99 | 0,41 | 0,02 |
| 13904870 | ribosomal protein S5 | RPS5 | 22 | 10,06 | 0,41 | 0,03 |
| 9845502 | ribosomal protein SA | RPSA | 62 | - | 0,41 | 0,06 |
| 32454741 | *serine (or cysteine) proteinase inhibitor, clade H, member 1 precursor* | *SERPINH1* | 46 | 0,77 | 0,41 | 0,01 |
| 11345462 | signal peptidase complex subunit 3 | SPCS3 | 20 | - | 0,41 | 0,03 |
| 4758140 | unnamed protein product | DDX6 | 54 | 1,04 | 0,41 | 0,06 |
| 4503015 | copine III | CPNE3 | 38 | 0,96 | 0,40 | 0,05 |
| 25453472 | eukaryotic translation elongation factor 1 delta isoform 2 | EEF1D | 60 | 0,10 | 0,40 | 0,01 |
| 15718687 | *ribosomal protein S3* | *RPS3* | 14 | 1,02 | 0,40 | 0,04 |
| 4507651 | *tropomyosin 4* | *TPM4* | 28 | - | 0,40 | 0,04 |
| 5453597 | F-actin capping protein alpha-1 subunit | CAPZA1 | 32 | 0,11 | 0,39 | 0,13 |
| 68160937 | tripartite motif-containing 25 | TRIM25 | 70 | - | 0,39 | 0,03 |
| 116063573 | *filamin A, alpha isoform 1* | *FLNA* | 250 | 0,92 | 0,38 | 0,04 |
| 4504041 | *guanine nucleotide binding protein (G protein), alpha inhibiting activity polypeptide 2* | *GNAI2* | 15 | 0,99 | 0,38 | 0,03 |
| 21361114 | solute carrier family 25 (mitochondrial carrier | SLC25A11 | 34 | 9,26 | 0,38 | 0,01 |
| 15991827 | *hexokinase 1 isoform HKI-R* | *HK1* | 102 | 0,95 | 0,37 | 0,03 |
| 30520310 | *metadherin* | *MTDH* | 64 | - | 0,37 | 0,05 |
| 15431290 | ribosomal protein L11 | RPL11 | 20 | 1,01 | 0,37 | 0,01 |
| 12025678 | *actinin, alpha 4* | *ACTN4* | 104 | - | 0,36 | 0,04 |
| 17158044 | *ribosomal protein S6* | *RPS6* | 28 | 0,99 | 0,36 | 0,03 |
| 31621305 | leucine-rich PPR motif-containing protein | LRPPRC | 157 | - | 0,35 | 0,03 |
| 4757732 | programmed cell death 8 isoform 1 | AIFM1 | 66 | - | 0,35 | 0,03 |
| 4501891 | *actinin, alpha 1 isoform b* | *ACTN1* | 35 | 0,94 | 0,34 | 0,01 |
| 21361416 | related RAS viral (r-ras) oncogene homolog 2 isoform a | RRAS2 | 23 | - | 0,34 | 0,03 |
| 4758086 | cysteine and glycine-rich protein 1 isoform 1 | CSRP1 | 20 | 0,85 | 0,33 | 0,04 |
| 61743954 | AHNAK nucleoprotein isoform 1 | AHNAK | 629 | 1,00 | 0,32 | 0,05 |
| 108773810 | leucyl-tRNA synthetase | LARS | 17 | - | 0,32 | 0,05 |
| 4504811 | junction plakoglobin | *JUP* | 81 | 11,23 | 0,31 | 0,03 |
| 5031815 | lysyl-tRNA synthetase isoform 2 | KARS | 68 | 1,04 | 0,31 | 0,03 |
| 156071459 | *solute carrier family 25, member 5* | *SLC25A5* | 35 | 0,98 | 0,30 | 0,06 |
| 19920317 | cytoskeleton-associated protein 4 | CKAP4 | 66 | - | 0,28 | 0,04 |
| 33620775 | kinectin 1 isoform a | KTN1 | 14 | 1,03 | 0,26 | 0,04 |
| 209862851 | plastin 3 | PLS3 | 16 | 0,01 | 0,26 | 0,02 |
| 71773415 | annexin VI isoform 2 | ANXA6 | 75 | 0,90 | 0,24 | 0,01 |
| 105990514 | filamin B, beta (actin binding protein 278) | FLNB | 278 | - | 0,23 | 0,03 |
| 116805322 | *gamma filamin isoform a* | *FLNC* | 291 | 0,75 | 0,23 | 0,04 |
| 4507813 | *UDP-glucose dehydrogenase* | *UGDH* | 55 | 9,11 | 0,23 | 0,03 |
| 16753203 | ubiquilin 1 isoform 1 | UBQLN1 | 62 | - | 0,22 | 0,01 |
| 15451856 | *caveolin 1* | *CAV1* | 20-22 | 0,96 | 0,21 | 0,04 |
| 7305053 | myoferlin isoform a | MYOF | 234 | - | 0,21 | 0,05 |
| 156104878 | *glutaminase* | *GLS* | 73 | 1,05 | 0,20 | 0,03 |
| 42734430 | polymerase I and transcript release factor | PTRF | 43 | - | 0,20 | 0,03 |
| 157694492 | MYB binding protein 1a isoform 2 | MYBBP1A | 133 | 1,12 | 0,20 | 0,14 |
| 63252913 | *gelsolin-like capping protein* | *CAPG* | 38 | 0,83 | 0,16 | 0,04 |
| 21071056 | *SWI/SNF-related matrix-associated actin-dependent regulator of chromatin a4 isoform B* | *SMARCA4* | 184 | 0,74 | 0,16 | 0,08 |
| 5453555 | ras-related nuclear protein | RAN | 24 | - | 0,07 | 0,11 |

^1^SD, standard deviation (n = 2)
